# The more it changes the more it stays the same: The French social space of material consumption between 1985 and 2017

**DOI:** 10.1111/1468-4446.12970

**Published:** 2022-07-19

**Authors:** Maël Ginsburger

**Affiliations:** ^1^ Center for Research on Social Inequalities (CRIS) Sciences Po|CNRS Paris France; ^2^ Center for Research in Economics and Statistics (CREST) GENES|CNRS Palaiseau France

**Keywords:** consumption, lifestyles, social change, social class, social space

## Abstract

The alleged homogenization of material consumption patterns in Western societies in the end of the twentieth century has been a central argument of scholars who predicted a general flattening of class inequalities. However, divisions in material consumption practices and their evolution have largely been neglected in studies of the social stratification of lifestyles. Drawing on six waves of the French Households Budget Surveys from 1985 to 2017 and Geometric Data Analysis, this article shows that the two main structuring oppositions in the French space of material consumption remained unchanged over 32 years. Those two divides are strongly but not exclusively associated with social class. The first persistently opposes integration with and exclusion from mass consumption. The second opposes connected and autonomous consumption styles. However, between 1989 and 2011, the practices associated with these divides have changed and households have experienced a major shift in their position toward the most integrated and connected poles. This study paves the way for comparisons to assess the permanence of those two polarities in material consumption—not only across periods, but also in different countries.

## 1 INTRODUCTION

Over the last 60 years, material consumption has been central to the debate about the decline, continuity, and transformation of class inequalities in Western societies. How the consumption of goods as diverse as automobile, refrigerators or beef has become more and more accessible for members of working classes in the post‐war period has been analyzed as key evidence of the declining structuring effect of class position on lifestyles (e.g., Zweig, 1961). Thus, in a “post‐traditional world” subjected to processes of individualization (Beck, 1992; Giddens, 1991), or in “post‐industrial” and “information” societies (e.g., Gershuny, 2003; Van Dijk, 2005), class divides would no longer structure the consumption of allegedly massified consumer goods and would vanish or shift toward flourishing areas of consumption such as services or information, rendering the appropriation of the material content of consumption increasingly fluid. However, such presumed changes in material consumption have not been submitted to a systematic empirical analysis. Based on the French case, this article relies on a social space approach to identifying and analyzing the main divisions organizing material consumption practices and the changes that have occurred in them since the late 1980s.

Scholars inspired by the practice theory have shown the benefits of studying material consumption items and practices related to equipment, travel, clothing, food and energy (Gronow & Warde, 2001; Warde, 2016), while massively neglecting the question of the unequal distribution of the individuals and households they recruit in the social space (Kennedy et al., 2015, pp. 13–14). Since the pioneering work of Bourdieu (2018), distinction in material consumption practices have largely been neglected in favor of those structuring cultural practices in the literature on lifestyles (Bennett et al., 2009; Flemmen et al., 2019; Weingartner & Rössel, 2019). This is especially true in France, where cultural boundaries were seen as particularly strong at the end of the 1980s (Lamont, 1992). This article aims at closing this gap by analyzing how, for the last 35 years, material consumption has also been affected by stable divides reflecting long‐lasting social and residential inequalities.

This article also intends to contribute methodologically to the study of social spaces in the long term (Coulangeon, 2013; Rosenlund, 2019). While empirical approaches to social space have resulted in a rich body of comparative work across countries (Atkinson, 2021a; Lemel & Katz‐Gerro, 2015), they rarely confront the question of social change.^1^ Such temporal variations are twofold, involving transformations in the space morphology and in the position of certain social classes or groups in the social space (Bourdieu, 1998, 2018). Some works focus on the study of trajectories, assuming a high degree of stability in the structure (Coulangeon, 2013), while others analyze both (Rosenlund, 2019; Weingartner & Rössel, 2019). I consider these two movements to be intrinsically linked: the stability of the structure of a social space must first be established in order to analyze the movements occurring within it. This article therefore aims primarily to analyze the stability of the space of material consumption and the changes in its main divisions. Beyond the empirical description of the relations between households and practices, to what extent do the axes of opposition emerging from the analysis of a social space constitute a rigid structure that reproduces itself and persists over time when generations and practices are renewed? Once this structure is established, the average movement of the whole population along those persistent divides will be described.

Focusing on the case of France and relying on six waves of the National Households Budget Survey between 1985 and 2017, I propose an innovative method based on Geometric Data Analysis to study the stability of the structure of a social space over time while documenting the movements of populations within it. I show that the space of material consumption styles is persistently organized by two divides: opposing integrated and excluded consumers, on the one hand; and connected and autonomous consumers on the other. Further, I demonstrate that the population globally has moved toward the active and connected poles. While Bourdieu has been widely criticized for focusing on reproduction and neglecting changes (Connell, 1983; Hobsbawm, 2008), this article shows the potential of a relational approach for understanding historical transformations by showing how substantial change can occur in a social space while the general structure of differences persists over time.

## 2 HOMOGENIZATION AND DIVISIONS IN MATERIAL CONSUMPTION

Increasing affluence in Western societies in the post‐war period has led social theorists to postulate a progressive disappearance of class‐structured inequalities in the consumption of material goods. In the 1960s, the notion of embourgeoisement (Zweig, 1961) described this alleged phenomenon of progressive homogenization of lifestyles and values, and of assimilation of former members of the working class into a large middle class. While rapidly criticized (Goldthorpe et al., 1971), the idea of a progressive flattening of class‐structured inequalities in consumption kept flourishing, as in Peter Saunders (1986) sociology of consumption or in sociological accounts of the presumed transition of our affluent Western societies from modernity to post‐ (or late) modernity (Beck, 1992; Featherstone, 2007; Giddens, 1991; Lash & Urry, 1987). In the latter theories, this alleged transition generates increasing fluidity of consumption patterns and the individualization of lifestyles through an “aestheticization of everyday life” (Featherstone, 2007), which materialize the narratives that help individuals to define themselves (Giddens, 1991).

Instead, divides opposing material consumption patterns according to social class could progressively be displaced by other divides—a view sustained in three different theoretical perspectives. The first revives an old economicist view that considers income to be the main structuring factor in consumption patterns (e.g., Engel, 1857). For Gartman, as prosperity grows, income hierarchy progressively becomes the almost unique structuring principle in material consumption, for which households regularly become differentiated according to the volume and intensity of acquisition and uses of goods and services (Gartman, 1991). In the second view, as social class loses its power, consumption patterns become differentiated by a multiplicity of other structuring inequalities—including age, gender, region and residential characteristics (Saunders, 1986). Changes in the content of—or access to—material products, such as technological or commercial innovations (IT hardware, new forms of motor vehicles, online sales), could amplify these divides, starting with the generational gap (Leach et al., 2013; Schmidt et al., 2014). In the third view, a more specific divide between a “middle mass” of included consumers—with access to employment and home ownership—and an “underclass” of excluded consumers progressively structures material consumption patterns (Bauman, 2007; Haupt, 2003; Saunders, 1986).

Empirical works on consumption in France in the second half of the twentieth century tend to confirm that the degree of integration to material consumption organizes consumption patterns (Daumas, 2018; Sirinelli, 2007; Vangrevelinghe, 1969). From the post‐war period until the 1990s, while some households became pioneers in the purchase of technological equipment, consumed a great deal of clothing, engaged in long‐distance travel and used considerable amounts of energy, the consumption habits of others remained frugal. The lifestyles of the latter group have been described as a residual outcome of the “society of scarcity” (Fairchilds, 1993), associated with poverty or distance from commercial areas and characterized by low consumption of fashion, energy, transportation, meat and durable goods (Boichard, 1958; Champagne, 2002; Chauvel, 1999). However, class inequalities still show up through this opposition. While the upper and middle classes appear to be at the forefront of these transformations (Daumas, 2018, pp. 360–387), and to embody the modern and integrated pole of material consumption, “excluded consumers” (Williams & Windebank, 2002) mainly comprise manual workers and employees, but also small farmers, unemployed and economically inactive people. Such works suggests that, even if transformed or even merged with an opposition in terms of inclusion to mass consumption, class‐based inequalities in material consumption have maintained in the second half of the twentieth century in Western countries such as France. This article aims to assess the extent to which structured inequalities in material consumption—such as those related to social class—have maintained or been replaced by lifestyle fragmentation or other divides, such as those relating to income or exclusion from consumption.

## 3 STUDYING MATERIAL CONSUMPTION THROUGH A LONG‐TERM SOCIAL SPACE PERSPECTIVE

For a large proportion of the members of Western societies, increasing affluence since the end of the post‐war period has been associated with a wider access to a number of commodities and consumer experiences. Some practices have been at the heart of the affirmation of a “material culture” (Gartman, 1991) in which the quest for comfort, practicality, cleanliness (Shove, 2003) and freedom (Trentmann, 2004) is conducted through intense and renewed forms of consumption. Previous works on practices related to cars (Coulangeon et al., 2015) or household appliances (Jacobsen, 2019) gathered those commodities under the umbrella of “material consumption”. In this article, I use the broader notion of *material consumption practices* to describe the set of practices that reflect forms of adhesion to—or rejection of—the material commodities valued in contemporary material culture. Many are ordinary, inconspicuous and apparently not very expressive (Gronow & Warde, 2001).

**FIGURE A1:**
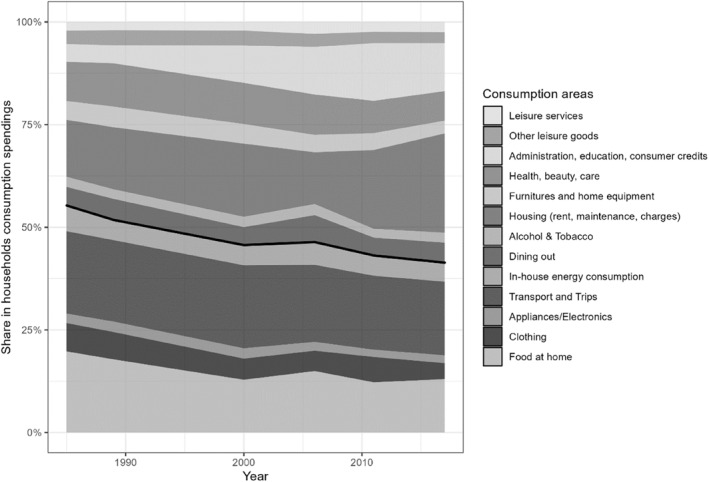
The share of the different consumption areas in households consumption spending in France, 1985–2017. *Source*: INSEE – BDF 1985, 1989, 2000, 2006, 2011, 2017.

I choose to focus on consumption practices that have long been integrated into material consumption culture, and that are related to food, household appliances, leisure electronic devices, transportation, energy and clothes. I leave aside education, health expenditure and services such as buying and listening to digital music or having meals delivered, which are typical of a recent evolution of consumer culture. Material consumption practices iconic of the “mass consumption” era (Urry, 1990) have remained central in the definition of contemporary consumption patterns. Indeed, spending dedicated to material consumption still represents a large share of household consumption budgets in France: 41% in 2017 (when limiting such spending to food at home, clothing, appliances/electronics, transport and in‐house energy consumption) despite a decline of 14 points since 1985, mainly in favor of spending dedicated to housing, consumption credits and administrative charges (see Figure A1). Such practices also exclude those related to cultural consumption, which have received more attention from scholars interested in the social stratification of consumption since the publication of Bourdieu's *Distinction* (2018).

Works inspired by the theory of practices have contributed to the study of material consumption practices (e.g., Shove & Southerton, 2000 on the use of the refrigerator), and have invited attention to the relations between practices (Shove et al., 2012). But they rarely consider issues of conflict, class or distinction when analyzing the social dynamics of consumption practices (Kennedy et al., 2015). For some authors, the allegedly massified material culture would not be subject to class distinctions, contrary to nonmaterial culture (Gartman, 1991); however, several authors have shown that such class‐based distinctions may affect material consumption. These studies focus on specific practices, such as those related to cars (Coulangeon et al., 2015) or food (Atkinson, 2021b), while others integrate some material practices in the definition of the space of lifestyles (Atkinson, 2021a; Flemmen et al., 2019). Finally, others focus mainly on expenditure patterns (Katz‐Gerro, 2003; Petev, 2011). In line with those previous works, I study divisions in material consumption and systematize their approach by considering a wide range of material consumption practices and the changes that may have affected such divisions in the recent period.

The notion of lifestyle proves particularly useful in binding typical configurations of practices, their statutory meanings and the social groups associated with them. I propose the notion of *material consumption styles* to refer to such configurations of material consumption practices, given that these material consumption styles are central elements of lifestyles but not equivalent to them.^2^ The approach in terms of social space has proven effective in the study of mainly cultural lifestyles (Bennett et al., 2009; Bourdieu, 2018; Flemmen et al., 2019). I also consider this analytical tool as a heuristic approach for studying the main divides across material consumption styles. Applying the social space framework to the study of material consumption also enables to compare the nature of the divisions occurring in such spaces and those frequently observed in cultural consumption (highbrow vs. lowbrow, engagement vs. disengagement, traditional vs. emergent) as well as the main inequalities on which they rely (such as age or capital composition).

Relying on a Bourdieusian framework to assess social changes might seem surprising, since many authors consider that a major flaw of Bourdieu's analysis is the paucity of his account of social change compared with the importance of social reproduction (Connell, 1983; Hobsbawm, 2008). Instead, I advocate that the social space approach is heuristic for understanding both change and stability because it carries a relational approach to structuring inequalities. In such an approach, classes or lifestyles are not fixed entities but rather are defined in relation to each other and coexist in socially structured spaces. As I will show, this approach allows me to tackle the way changes affecting both practices and individuals may occur, while the general structure of differences persists.

This article therefore aims to analyze the space of material consumption styles in France over the last 3 decades to assess (1) the degree of stability and change that has affected the main structuring oppositions in material consumption styles; and (2) the strength and the nature of class‐related inequalities in such oppositions.

## 4 METHOD

### 4.1 Data and measures

This research is based on six waves of the French Household Budget Survey undertaken by the French National Statistical Institute (INSEE): 1984–85, 1989, 2000–01, 2006, 2011 and 2017. These surveys interviewed between 9000 and 12,000 metropolitan households randomly drawn from the census master sample to collect information on their resources and expenditures. The net response rates vary between 49 and 54%. The aggregated sample covers 63,983 households. The data collection relies on diaries where respondents report each expenditure during a week (two before 2011) and face‐to‐face interviews to tackle residential, financial and social characteristics, periodic or exceptional expenditures, and food produced for the household's own consumption. Since 2011, this dataset has been merged with information on income from administrative sources.

A total of 28 categorical variables were computed, referring to consumption practices, habits and material possessions in five different areas: food consumption and supply; electric and electronic devices; home energy consumption; clothing; and transportation (Table 1).^3^ These variables cover various areas of material consumption, and are available for every survey wave while including practices far more frequent in the beginning (food production for one's own consumption) or in the end (IT devices purchase) of the period. Although monetary data are the first target of the French Household Budget Surveys, the amounts spent in a consumption category represent imprecise information when studying the nature and volume of acquisitions. Indeed, the same amount spent can reflect high prices or high volumes. Further, consumption is not only about acquiring goods and services but also dealing with their appropriation and appreciation (Warde, 2016). I therefore introduced variables to measure—within the limits of the data—repairing or maintenance practices, production for one's own consumption, and nature and intensity of energy use. When volumes were not directly available or estimable using price time series, I replaced them by spending amounts (in euros of 2011) per consumption unit when prices varied poorly across time and items (e.g., bottled water) or when the measure gathered heterogenous services (e.g., public transportation), or by dedicated budget shares when there were strong price differences across products depending on quality (e.g., beef).^4^

**TABLE 1:** The 28 active variables related to material consumption practices

Variables	Categories
**Share in in‐house food budget**	
Beef and veal	4
Pork and poultry	4
Butter and cheese	4
Vegetables	4
Cereal products	4
**Diversity of food produced for own consumption (out of five categories)**	3
**Spending on bottled water per consumption unit**	4
**Owning**	
Freezer	2
Dishwasher	2
Washing machine	2
**Purchasing in last year**	
IT devices	2
Audio/video/camera devices	2
Household appliances	2
**Acquiring second‐hand devices**	4
**Extending devices lifespan through reparation services or resale**	2
**Purchasing in last 2 months (per person)**	
Shoes	3
Pants, dress or skirt	3
Leisure/outwear clothes	2
**Repairing or maintenance practices: Repairing or cleaning services, haberdashery products, spending on textiles**	2
**Type of energy used for heating**	5
**Spending in piped water**	4
**Estimated volume in non‐renewable energy per consumption unit** (Estimation and sum of the Net Calorific Value [NCV] of the annual volume of electricity, gas, fuel oil, wood and coal bought [relying on the French Pegase dataset—SOeS])^a^	5
**Number of cars**	3
**Diesel engines**	2
**Volume of oil consumed** (relying on French Pegase dataset—SOeS)	4
**Spending in public transportation per consumption unit**	4
**Trips in the last year**	
Abroad	3
Domestic^b^	4

^a^ I use Multiple Imputation by Chained Equation to separate gas and electricity spending when those are paid jointly (28,205 households). *Pegase* (for “*Pétrole, électricité, gaz et autres statistiques de l’énergie*”) is a database produced by the statistical department of the Department of Environment and Energy aimed at publicizing evolution in prices—for households and companies—of the main energies.

^b^ I use Multiple Imputation by Chained Equation on households from the 2006 wave to estimate the number of trips during the last year, this information only being available for the last 6 months (10,240 households).

I classically use three indicators of social class: occupation, income, and education (e.g., Flemmen et al., 2018). I therefore analyze the ways in which different forms of capital—cultural and economic—interplay and contribute to position in the social space (Bourdieu, 2018; Savage, 2000). But since such capital dotation only poorly estimates the relations of powers and domination bounded to the place on labor market (see Flemmen, 2013), I also consider occupational position. Class, like the other variables, is analyzed at the household level, considering households' living standard percentiles, highest diploma and occupational category, relying on the French occupational scheme at the household level (Amossé & Cayouette‐Remblière, 2019).

### 4.2 Empirical strategy

This work relies on the use of Geometric Data Analysis (GDA) methods (Le Roux & Rouanet, 2004), which enable identification of factors of opposition or similarity among a set of variables within a population and inductively distinguish and prioritize the main logics (named axes or dimensions) segmenting that population according to those variables, producing a map or space. GDA tools also enabled me to project supplementary variables useful for interpreting the dimensions in the space, such as social or residential characteristics, or to project supplementary individuals as long as they are also characterized by all the active variables (Bennett et al., 2009; Coulangeon, 2013).

Few empirical works relying on a social space approach measure the temporal changes occurring in the spaces they study (but see Coulangeon, 2013; Rosenlund, 2019; Weingartner & Rössel, 2019). The method traditionally used involves building a space (using Multiple Correspondence Analysis [MCA]) on the individuals from the first year and projecting individuals from posterior years as supplementary. As indicated in Table 2, this method does not account for the changes affecting the dimensions of the space. Furthermore, variations in individuals' position across years are measured on the axes from one year only. In case of low similarity between the annual spaces, the individuals from posterior years are positioned along a divide that no longer concerns them. And in case of high similarity between the annual spaces, such an approach considers the space from the first year as a proxy for the “long‐term space” instead of actually comparing individuals' position in a space closer to the “long‐term space”—that is, a compromise space that ignores annual specificities. I argue that this method, when not complemented by additional analyses (e.g., Rosenlund, 2019), fails to adequately measure and analytically distinguish changes into the structure of spaces and changes into individual positions in an allegedly stable social space. Other methods, such as performing a single MCA in the full sample containing all the individuals, also have severe limitations (see Table 2). Moreover, the data used in this study are not panel data, which prevent from using more sophisticated methods such as Multiple Factor Analysis. I therefore propose a two‐step analytical strategy aimed at analyzing changes affecting both a social space structure and individuals' position inside it, which can prove useful when the data are not panel data but the set of variables is homogeneous across survey years.

**TABLE 2:** Four empirical strategies for analyzing social spaces across periods

	Multiple Correspondence Analysis on a single sample year	Multiple Correspondence Analysis on the full sample	Multiple Factor Analysis on the full sample	Multiple Correspondence Analysis on each sample year followed by PCAs on common axes.
Description	Comparing individuals' position across time in a single MCA with individuals from 1 year as active and individuals from posterior and/or prior years as supplementary	Comparing individuals' position across time in a single MCA with individuals from all sample year as active	The same individuals are characterized by x groups of the same n variables corresponding to the x survey years. Separated analyses on each annual group of variables produce “annual spaces” while a compromise “long‐term” pace between these spaces is computed.	(1) The x annual groups of individuals are analyzed separately using MCAs. Individuals from other years are projected as supplementary in each annual space. The stability of the main dimensions is assessed. (2) Calculation of a compromise “long‐term” space, using PCAs on each set of common dimensions.
Strengths	‐ A one‐step process. The divides are easy to interpret as they are only associated with 1 year. ‐ Widely used (see Coulangeon, 2013; Purhonen et al., 2021; Rosenlund, 2019)	‐ A one‐step process. ‐ All the individuals contribute to the divides.	‐ A one‐step process. ‐ Produces both annual spaces to assess changes in their structure and a compromise “long‐term” space to assess changes in individual positions.	Produces both annual spaces to assess changes in their structure and a compromise “long‐term” space to assess changes in individual positions. The relevance of the latter depends on the degree of similarity of the formers, which can be assessed.
Limitations	‐ Requires the same variables each year. ‐ Changes in individuals' position are measured on divides accounting for 1 year only. ‐ Changes in the structure are ironed out.	‐ Requires the same variables each year. ‐ Changes in the structure are ironed out. ‐ The divides confuse divides in each annual subsample and temporal changes during the period studied.	Requires panel data.	‐ Requires the same variables each year. ‐ Complexity of a two‐step process.

For each of the six waves (1985, 1989, 2000, 2006, 2011, 2017), I performed an MCA^5^ on all metropolitan households and the 28 variables. The few residual missing values were imputed using the MIMCA method (Josse & Husson, 2016) and do not contribute to the construction of the dimensions. I focus on the first two dimensions of each of the six MCAs, which—depending on the year—summarize between 78 and 84% of the total modified variance rate (Le Roux & Rouanet, 2004, p. 200) (see Table A1).

For the MCA on the 28 consumption variables and the 11,977 households of the 1985 wave, I named “space of material consumption in 1985” the plane produced by the intersection of the first two dimensions (see Figure A3). I then projected the 52,006 households from the other waves (1989 to 2017) onto each of the first two axes. In this way, each of the 63,983 households—whether they contributed to the construction of the space of material consumption in 1985 or not—had a coordinate on each of the two dimensions structuring the space for that year. I did the same for the five other waves (Figures A4, A5, A6, A7, A8). Therefore, each household had a coordinate on the two axes structuring each of the six annual spaces of material consumption (from 1985 to 2017).

It was thus possible to compare each household's position in the space of a year and in the space of the following year, and to assess the extent to which the axes of the annual spaces opposed the same households and the same consumption practices. I assessed the stability of the structure of the space of material consumption by comparing both the position of the 63,983 households in all annual spaces and the coordinates of the 89 active categories in such spaces. I used Pearson correlation coefficients to assess the similarity of households' coordinates on the axes from each annual social space. I did the same for active categories. A high correlation coefficient (greater than 0.8) between coordinates on axis 1 of year *n* and axis 1 of year *n* + *i* indicated that the two annual axes reflected the same divide, insofar as they mainly opposed the same households and the same consumption practices.

**FIGURE A2:**
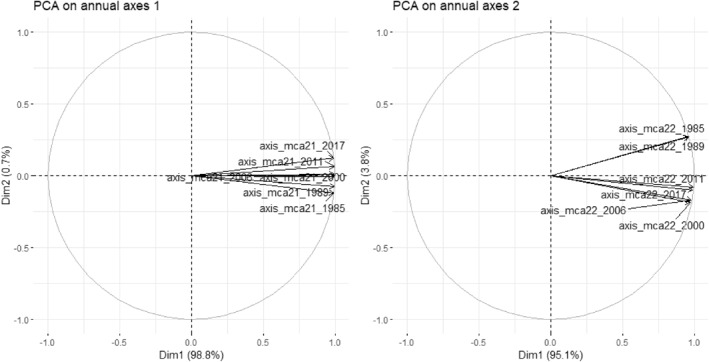
PCA correlation circles on the two sets of axes.

**FIGURE A3:**
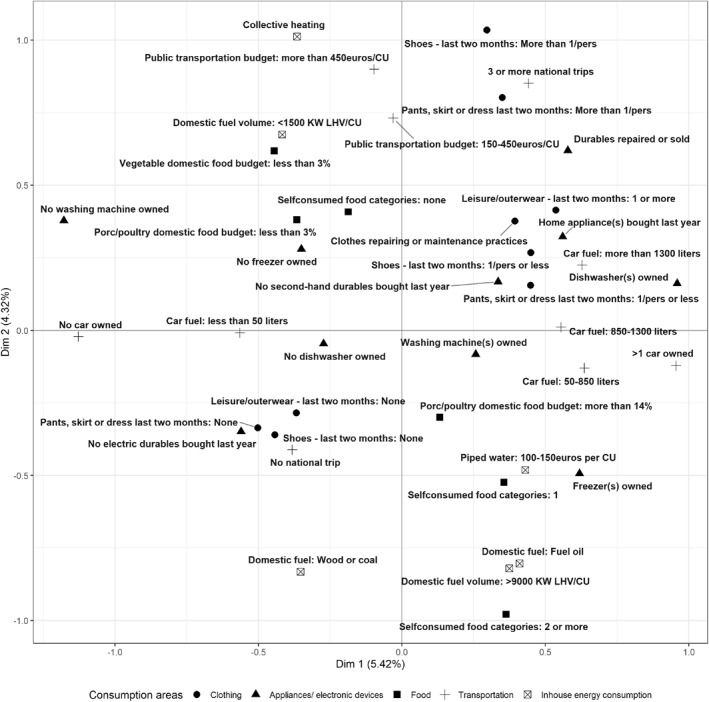
The space of material consumption in 1985: Main active categories. The categories projected have contributed strongly to the construction of at least one of the two axes, that is, their contribution is above the average contribution.

**FIGURE A4:**
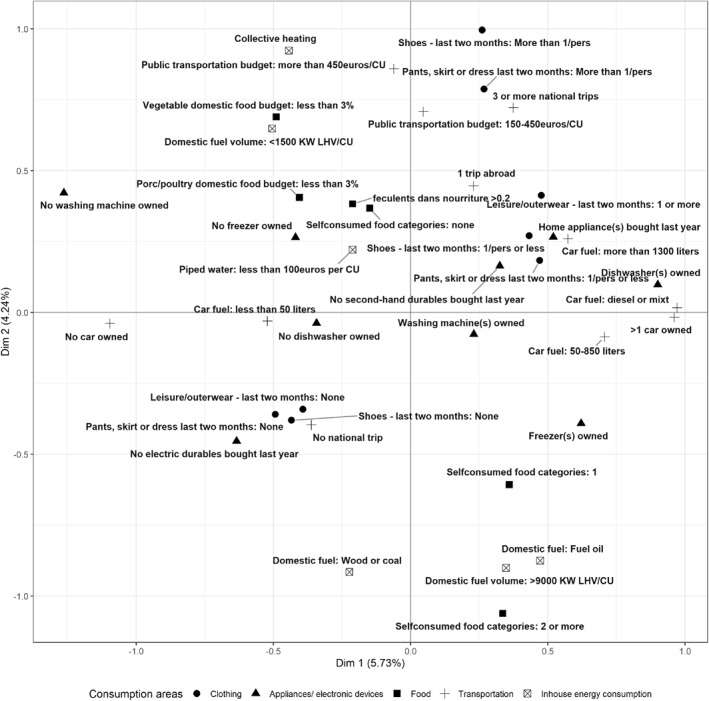
The space of material consumption in 1989: Main active categories. The categories projected have contributed strongly to the construction of at least one of the two axes, that is, their contribution is above the average contribution.

**FIGURE A5:**
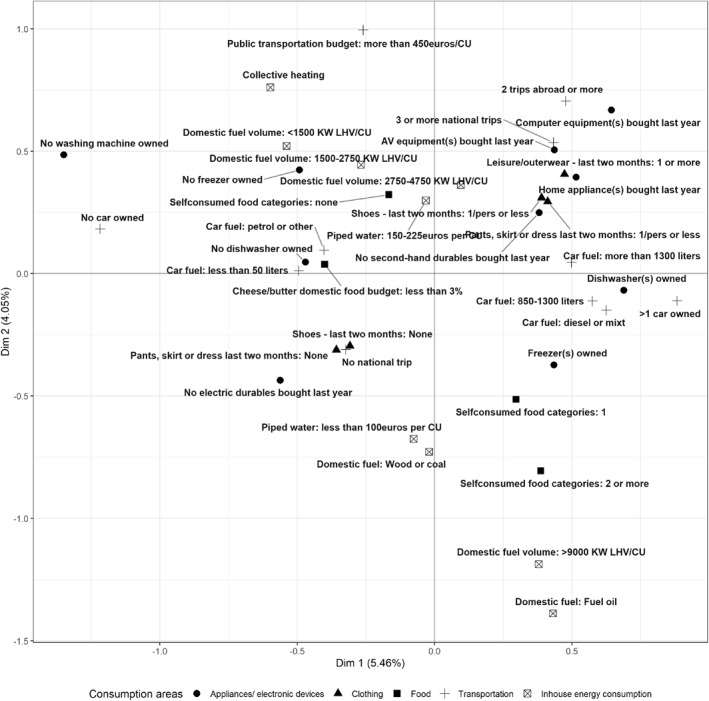
The space of material consumption in 2000: Main active categories. The categories projected have contributed strongly to the construction of at least one of the two axes, that is, their contribution is above the average contribution.

**FIGURE A6:**
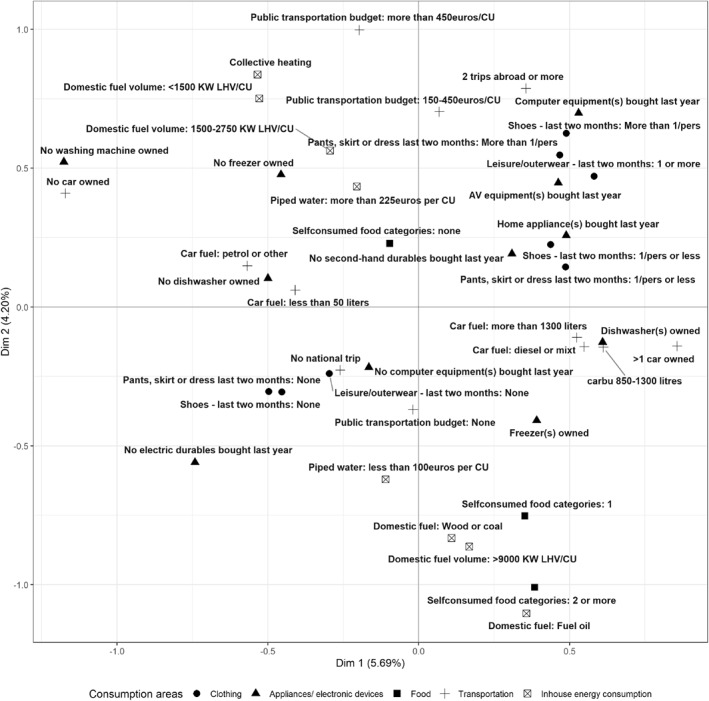
The space of material consumption in 2006: Main active categories. The categories projected have contributed strongly to the construction of at least one of the two axes, that is, their contribution is above the average contribution.

**FIGURE A7:**
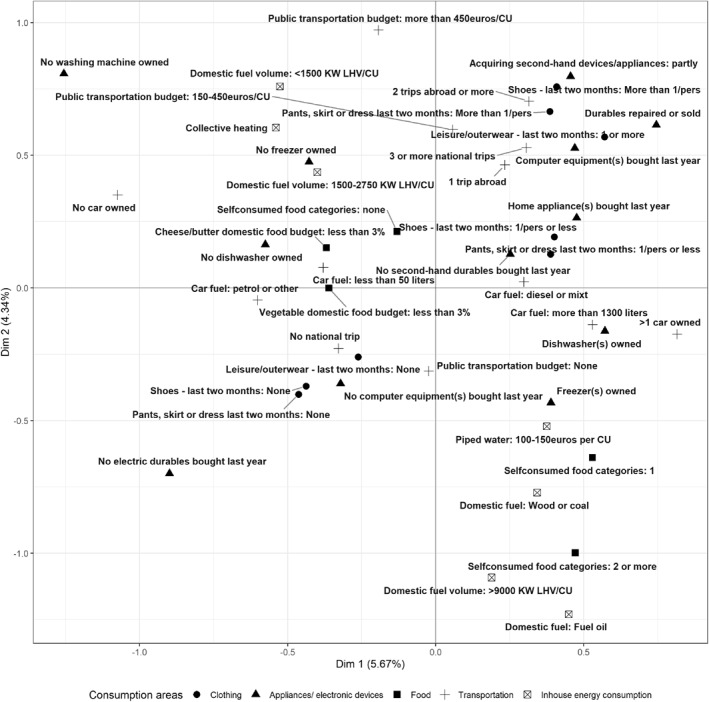
The space of material consumption in 2011: Main active categories. The categories projected have contributed strongly to the construction of at least one of the two axes, that is, their contribution is above the average contribution.

**FIGURE A8:**
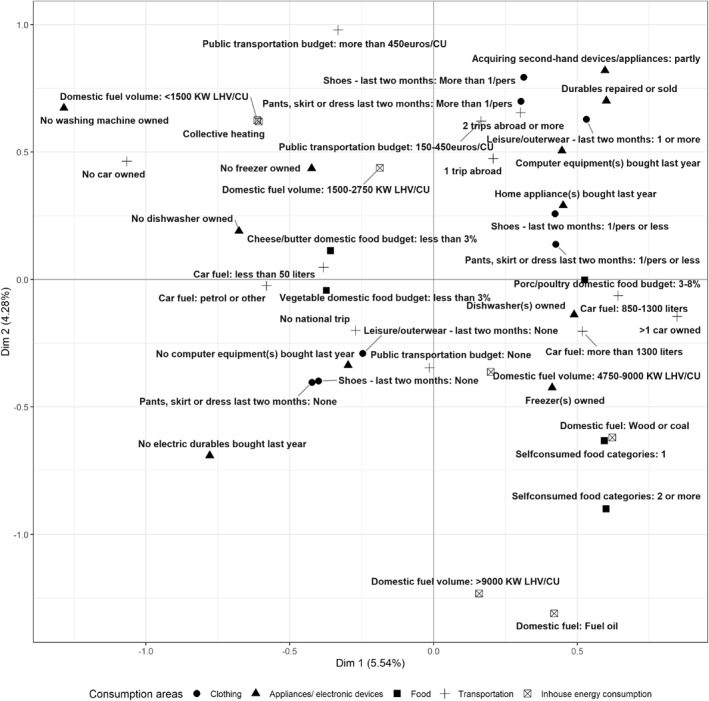
The space of material consumption in 2017: Main active categories. The categories projected have contributed strongly to the construction of at least one of the two axes, that is, their contribution is above the average contribution.

The axes observed across different waves are strongly similar, as reflections of the same latent long‐term axes. I therefore analyzed the plane produced by the intersection of these two long‐term axes as the “long‐term space of material consumption” (between 1985 and 2017). From the set of axes 1 from the six annual spaces, I identified the long‐term axis 1 using a Principal Component Analysis on the 63,983 households and the six variables measuring their positions on each annual axis 1 (Figure A2). This method identifies the main factors common to a set of numerical variables, in this case the highly correlated annual axes 1 for which each household has a coordinate. These variables are highly correlated with each other, as I have previously shown using Pearson's correlation coefficient between households' and categories' coordinates on each annual axes 1. I therefore used the first factor of the PCA, which summarizes almost all the inertia, as a compromise axis 1 of all annual axes 1 and I named this first factor “first dimension of the long‐term space of material consumption”. I proceeded in the same way for each annual axis 2 to distinguish the second long‐term dimension^6^ (Figure A2).

I then analyzed the changes in the association of certain practices with the structure of the space of material consumption to show how some practices' disappearance or scattering was relayed by the unequal diffusion of others in maintaining the same divides. I compared, across waves, the contribution of the different categories to the construction of each of the two axes as well as their frequency and coordinates in each annual social space. Contributions measure the influence of each category on an axis's definition, while coordinates measure their association with one or the other pole of the axis (see Table A3). Since the contribution of a category to an axis is proportional to its relative frequency and its coordinate on this axis (Le Roux & Rouanet, 2004, p. 196), analyzing the variations in categories' frequency and coordinates on each axis proves useful for providing a better understanding of the evolution of their contributions.

Finally, I documented the average movement of the population along the long‐term axes and showed how these were tied to generational changes. Since the households being sampled in each survey wave are not the same, this method does not track changing positions of households per se in the space, but rather studies movements of households in general.

## 5 RESULTS

### 5.1 A stable social space

**FIGURE 1:**
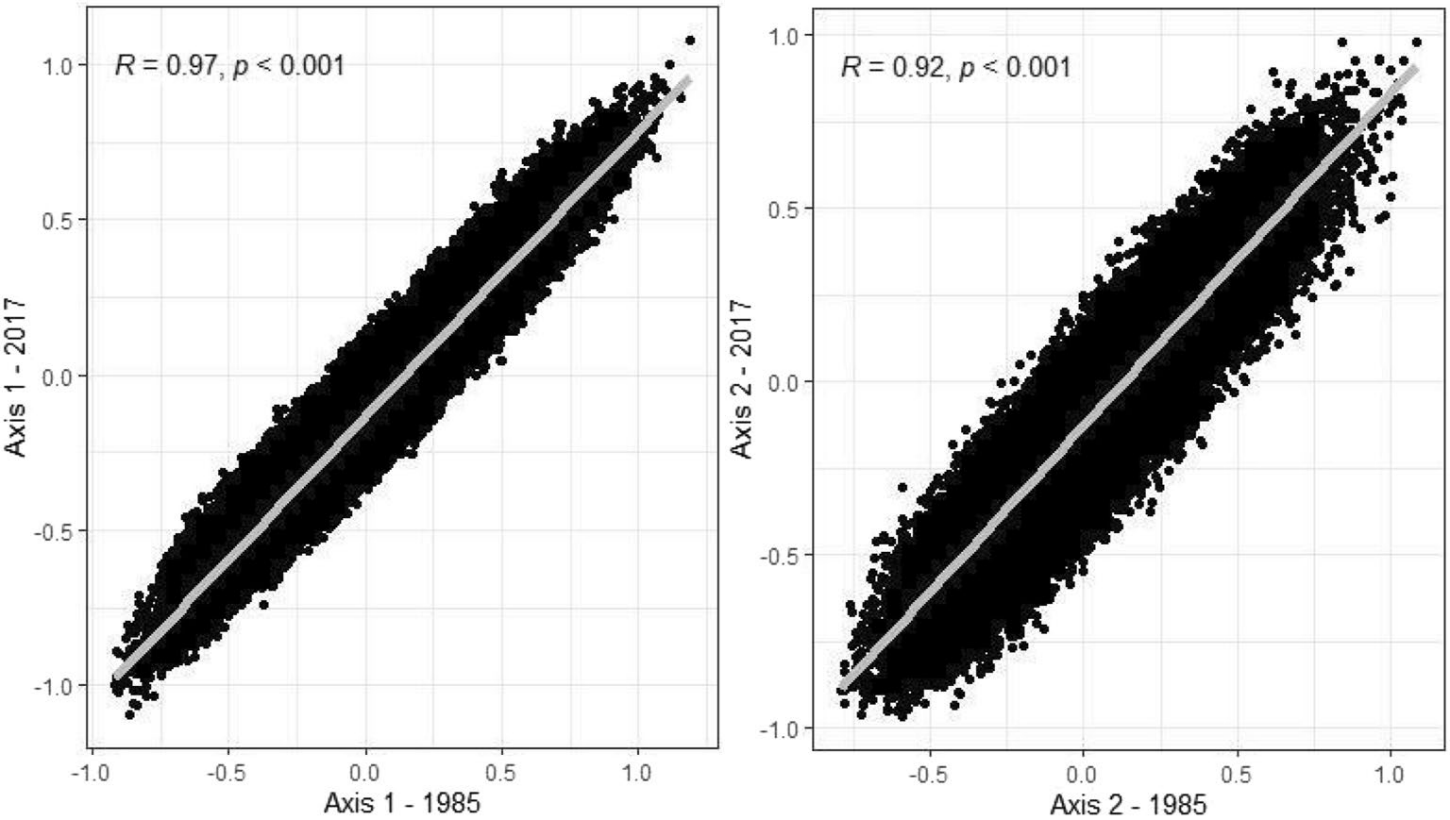
Correlation between the axes of the spaces of material consumption in 1985 and 2017. On the left side of this figure are plotted the coordinates of all 63,983 households on the axes 1 from the spaces of material consumption in 1985 and 2017. On this right side are plotted the coordinates of all households on the axes 2 from the spaces of material consumption in 1985 and 2017.

The structure of the space of material consumption has been stable over the past 30 years, organized along two main, largely identical axes. The structures of the spaces in 1985 and 2017 are extremely close (Figure 1), with a linear correlation coefficient of 0.97 between households' coordinates on both annual axes 1 and 0.92 between households' coordinates on both annual axes 2. More broadly, the correlation coefficients between annual axes 1 are always higher than 0.8, showing the strong similarity over the survey waves in the hierarchy between households along this first axis. The same conclusion applies to annual axes 2 (Table A2).^7^

**FIGURE 2:**
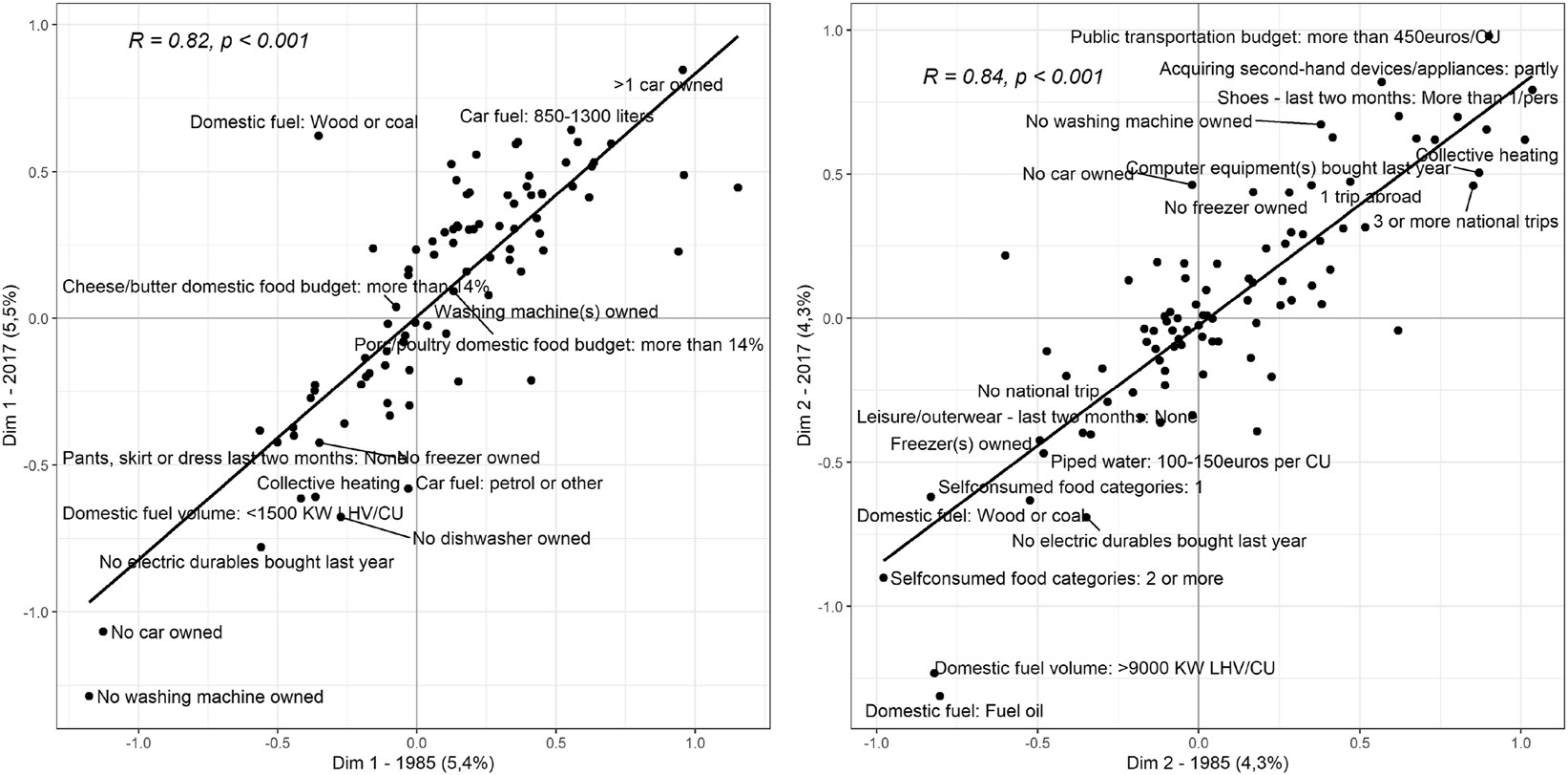
Correlation between the axes of the spaces of material consumption in 1985 and 2017—categories' coordinates. On the left side of this figure are plotted the coordinates of all 89 categories of the active variables on the axes 1 from the spaces of material consumption in 1985 and 2017. On this right side are plotted the coordinates of all categories on the axes 2 from the spaces of material consumption in 1985 and 2017.

When looking at the position of the active categories in that space, I find a similar result (Figure 2). The Pearson correlation coefficient is 0.82 between the coordinates of the categories on both annual axes 1 and 0.84 between households' coordinates on both annual axes 2. For categories too, the correlation coefficients between annual axes 1 (and between annual axes 2) are always higher than 0.8 (Table A3). Nonetheless, the coefficients are slightly lower than for households' coordinates because some categories—which contribute weakly to the axes—move across the space during the period studied (such as “Domestic fuel: wood or coal” for axis 1 and “Piped water: 100–150 euros per CU” for axis 2). I analyze those changes in Section 5.3.

**FIGURE 3:**
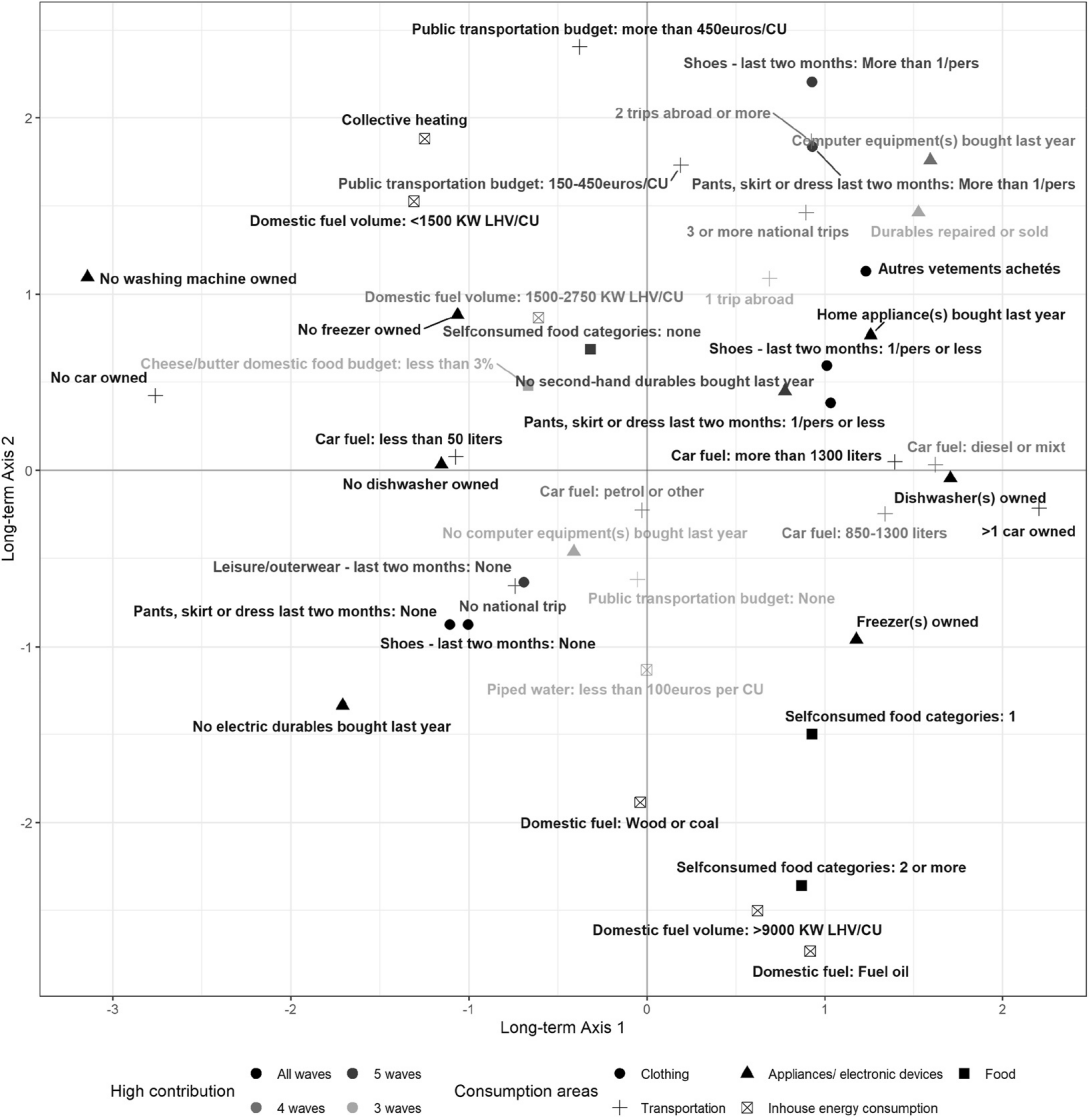
The long‐term space of material consumption: main active categories. The categories projected have contributed strongly contributed (more than the average contribution) to the construction of one of the two axes in at least three of the six annual spaces of material consumption. The contributions of each category to each annual axis are displayed in Tables A6 and A7.

Relying on the observation of the stability of the space of material consumption styles, I analyze the long‐term space—constructed by crossing long‐term axes 1 and 2^8^ determined from annual axes 1 and 2—and display the position of the highly contributing active categories the most stable across annual spaces (Figure 3, see Table A8 for details).

On the left side of the first axis are located households excluded from the consumption of a large proportion of goods and services. They are poorly equipped (no automobile or washing machine) and are low consumers of clothes, electrical and electronic devices, non‐renewable energy and vegetables. On the right side are households that are highly equipped, often owning multiple cars with diesel engines. They do not necessarily buy new or many objects: buying second‐hand devices, producing food for their own consumption or purchasing a moderate amount of clothing are also frequent practices among integrated consumers. Rather than measuring consumption intensity, on the basis of which households would be located according to the degree of prodigality of their consumption and their volume of purchases, this axis measures integration into mass consumption, opposing excluded consumers to well‐equipped households with average to intense consumption.

On the positive side (the top) of the second axis are households that make heavy use of public transport, travel intensively nationally, consume a lot of clothing and IT items, and extend the life of durable goods through second‐hand markets. Washing machines and freezers are rare and collective heating is common.

At the bottom, consumers are characterized by their high domestic energy consumption, especially fuel oil, wood and coal. They don't spend much on tap water (due to independent water supply sources), produce diverse types of food and, while highly equipped with freezers, their consumption of other durable goods (clothing or electronic and household devices) is low. Closer to self‐sufficiency, households on this side depend less on the market to renew clothes or devices, and on collective energy or transportation. This axis contrasts households with outward‐looking but also shared forms of consumption (in terms of transportation, heating systems, durable maintenance), with households with more autonomous and local consumption. The opposition between modern (at the top) and more traditional (at the bottom) forms of consumption also appears along this divide. We use the term “connected” to describe the form of consumption typical of the upper side of the space since it refers both to the high inclusion of these consumers into networked forms of consumption (of transportation, energy, second‐hand market) and to their affinity with modern technologies. We use the term “autonomous” to qualify traditional, localized and individualized patterns of consumption typical of the bottom side of the space. This axis thus opposes connected to autonomous consumption styles.

Those two orthogonal axes show that integration into the world of goods does not go hand in hand with outward‐looking, connected consumption.

### 5.2 Divides in material consumption and social and residential inequalities

**FIGURE 4:**
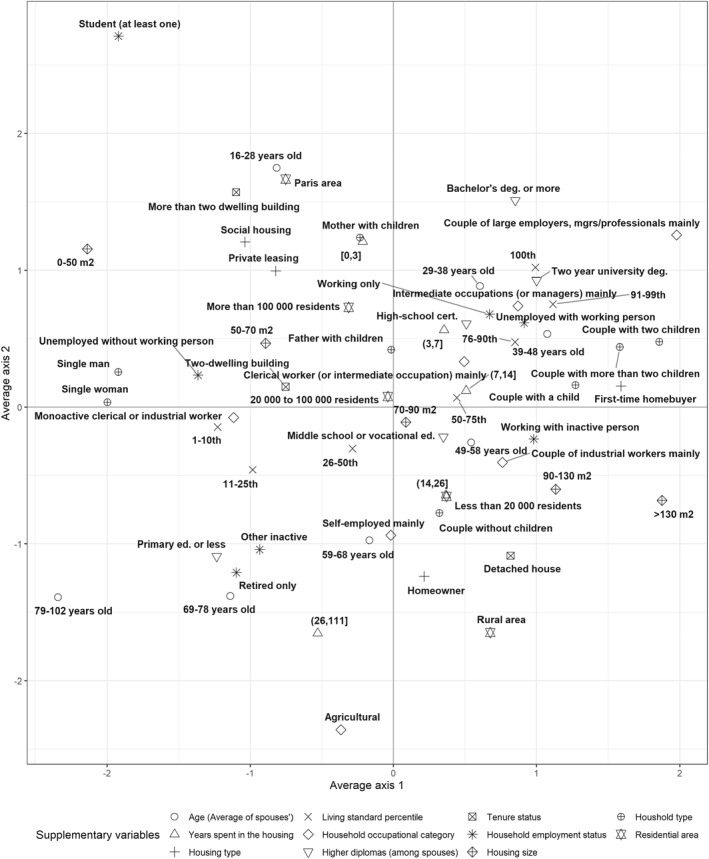
The long‐term space of material consumption: supplementary variables.

The projection of 11 supplementary socio‐demographic and residential variables in the long‐term space of material consumption styles helps us to better understand the social mechanisms at work in the two structuring divides (see Figure 4; see also Table A9 for coordinates and typicality tests).

The axis of integration into mass consumption mirrors divides in terms of class (diploma, living standard, occupation), age, household size and residential characteristics. Households excluded from mass consumption are mainly young or elderly, and often single persons or single‐parent families with low incomes. They are more likely to live in small (less than 50 m^2^) rented accommodation, generally in urban areas. Their members are most often not working, generally because they are students, pensioners, unemployed or for other reasons. Exclusion from mass consumption thus primarily concerns households whose positions in the labor, matrimonial or residential markets are particularly precarious. Conversely, integrated consumers are mostly well off, educated and employed households, especially managers. They are most frequently in the middle of their life cycle (39–48 years), living with at least two children in a large, recently acquired house.

The axis opposing connected and autonomous consumers overlaps with divides in terms of age, housing, occupation and education. Connected consumers are found mainly in young, highly educated households—particularly students or executives—living in large cities. Certain social or residential characteristics indicate possible forms of precariousness among some of these households (small size of housing, social housing, frequency of single mothers), while others, starting with the over‐representation of the 10% highest living standard on this side of the space, indicate the affluence of certain households. On the autonomous side (bottom) of the space are over‐represented farmers and older people, and more generally households that have been living for more than 25 years in owned houses located in rural areas. This second divide reflects an opposition that can be assumed to be generational, heightened by different relationships with space and different levels of cultural capital.

Income appears to be irregularly associated with both divides. An increase in income within the bottom 75% of living standards distribution is associated mainly with more integrated consumption, while an increase in the top 25% is associated mainly with more connected consumption.

### 5.3 The practices turnover

If the two major divides have persisted for more than 30 years, it is because certain practices have taken over from others in generating these divides. I measure such changes by looking at the evolution of categories' frequencies, and their contributions and coordinates on each axis along the 32 years (see Tables A4, A5 and A7 for details). Table 3 summarizes and provides examples of the five main changes in the space: quantitative decline; scattering; quantitative diffusion; concentration in one area of the space; and inversion in the association with an axis.

**TABLE 3:** The five main types of changes in practices association with the two main dimensions of the space of material consumption

	Quantitative decline	Scattering	Quantitative diffusion	Concentration	Inversion
Measure	Decreasing contribution		Increasing contribution		Inversion of the sign of coordinate
	… associated with strong decrease in frequency	… associated with strong decrease in absolute coordinate	… associated with strong increase in frequency	… associated with increase in absolute coordinate	
Main categories on axis					
1	No washing machine No car Car fuel 50–850 L	Freezer Washing machine Car fuel <50 L	IT devices bought Car fuel: Diesel or mixture	Car fuel: no diesel No dishwasher No IT device bought	Domestic fuel: wood or coal Piped water: more than €225 per CU Car owned: one
2	Food produced for own consumption: two categories or more	No food produced for own consumption No domestic trip Three domestic trips or more	IT devices bought Second‐hand durables bought Public transportation budget:>€450	No IT device bought No public transportation budget No leisure/outwear clothes	Car fuel: more than 1300 L Vegetables domestic food budget: more than 14% Piped water: more than €225 per CU

Practices contribute less to the generation of one axis when they become rare or less concentrated on one side of it. The quantitative decline or disappearance of certain practices greatly weakens their contribution to the existence of certain divides, since they no longer target more than an extremely small part of the material consumption style that they used to participate in defining. Thus, while the production of more than two categories of food for one's own consumption is still relatively frequent in 1985 (about 20% of households), and contributes relatively highly to the divide between connected and autonomous consumers (it explained 7% of the position on the axis), the decrease in its frequency (8% in 2017) makes it also very rare among households close to the autonomous side of the space (explaining only 2.5% of the variance on axis 2 in 2017). Moreover, some practices have become scattered—that is, less associated with one or the other of the two divides studied. This is the case for traveling nationally, which in 1985 is very divisive and contributes strongly to the connected/autonomous opposition, while in 2017 it has become a very widespread practice among French households. The categories “three domestic trips or more” and “no domestic trip” become much less typical of each side of the second axis (the coordinates go from 0.85 and—0.41 in 1985 to 0.46 and—0.2 in 2017).

Conversely, some practices experience movements of concentration on one pole of the axes studied or of large quantitative diffusion, leading them to contribute more strongly to such social divisions. For example, on the axis of integration to consumption, not having a dishwasher contributes more and more (from 1.76% to 5.69%). Normal among all households in 1985, the practice of washing dishes exclusively by hand becomes increasingly concentrated in the excluded side of the space, as dishwashers become particularly common among consumption‐integrated households. Meanwhile, the purchase of IT devices has undergone a phenomenon of massive quantitative diffusion, with a multiplication by 17 of the number of households having made at least one IT purchase during the last year. While impacting households in the excluded or autonomous poles of the space of material consumption, this diffusion has nevertheless taken place mainly in its most integrated and connected area, which becomes increasingly defined by this strong appropriation of IT devices. The contribution of the category “IT purchase” to both axes increases from 0.91% and 0.65% to 2.36% and 3.92% respectively between 1985 and 2017.

Technological changes and the appropriation of new social meanings can profoundly modify the nature of certain practices, reversing their association with an axis. A notable example is coal and wood heating, associated with the excluded area of the space in 1985 (−0.35), and becomes very strongly associated with the integrated area in 2017 (0.62), even though this type of heating energy remains rare overall. The spread of central heating with wood, together with the disappearance of coal, might change the meaning of such a practice, perhaps reflecting an increasingly modern, ecological and desirable heating practice in the recent period.

### 5.4 Toward more integrated and connected consumption styles

**FIGURE 5:**
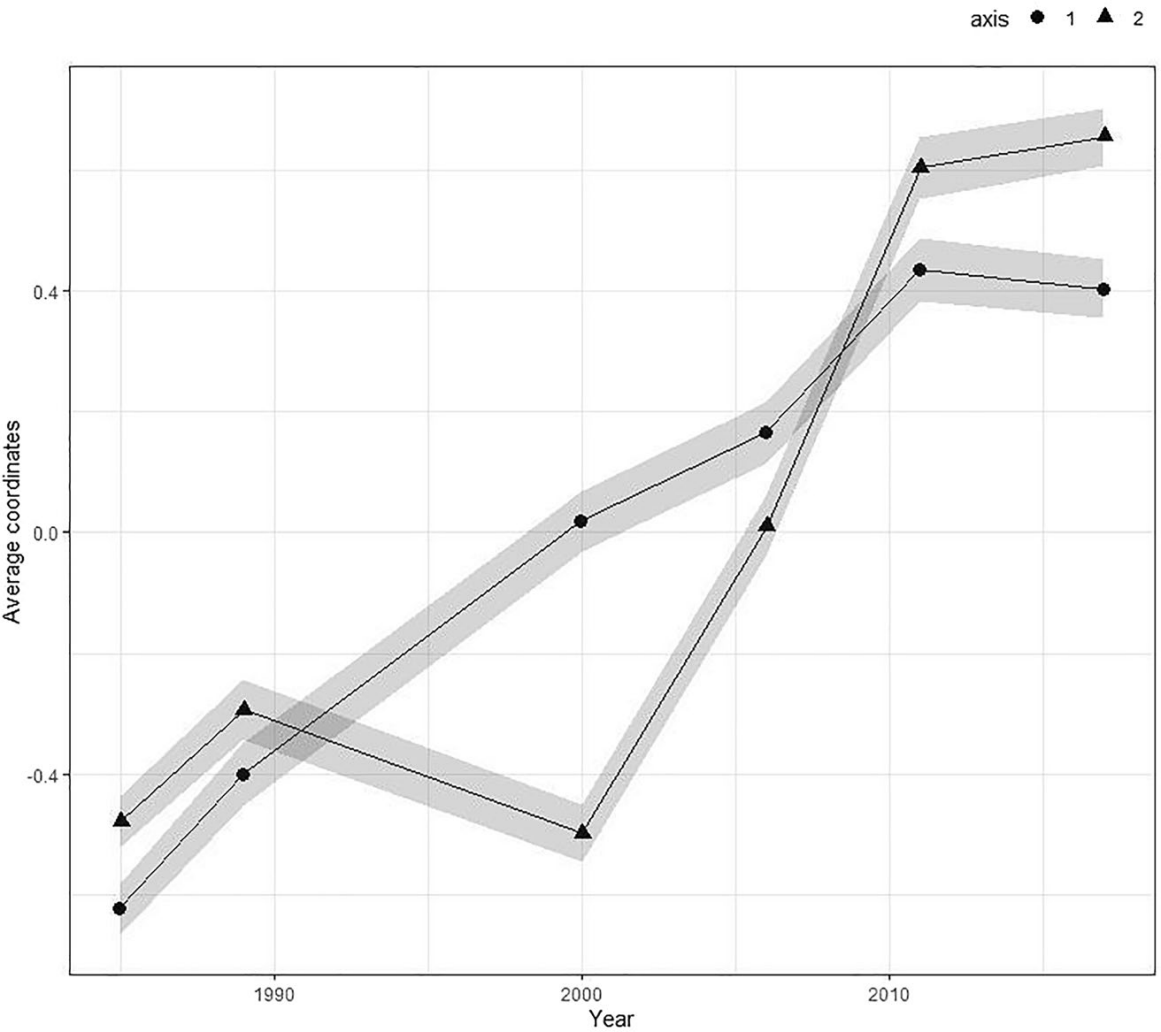
Average coordinates of French households on axes 1 and 2 of the long‐term space of material consumption between 1985 and 2017.

The persistent structure of the space over the years does not imply that French households occupy, in general, the same average position in 2017 as in 1985 (Figure 5). Once again, the result of the stability of the structure should not lead one to consider that nothing has changed since 1985.

French households have moved significantly in this space in association with generational changes. Households have moved from the excluded to the included area of the first axis between 1985 and 2011, with 1985 households having an average coordinate of −0.5 while 2011 households have an average coordinate of just over 0.4. This reflects the development of domestic and automotive equipment, and clothing and energy consumption. Between 2011 and 2017, however, there is a stagnation in the position, suggesting a break in the overall movement of households toward a more integrated consumption style.

**FIGURE 6:**
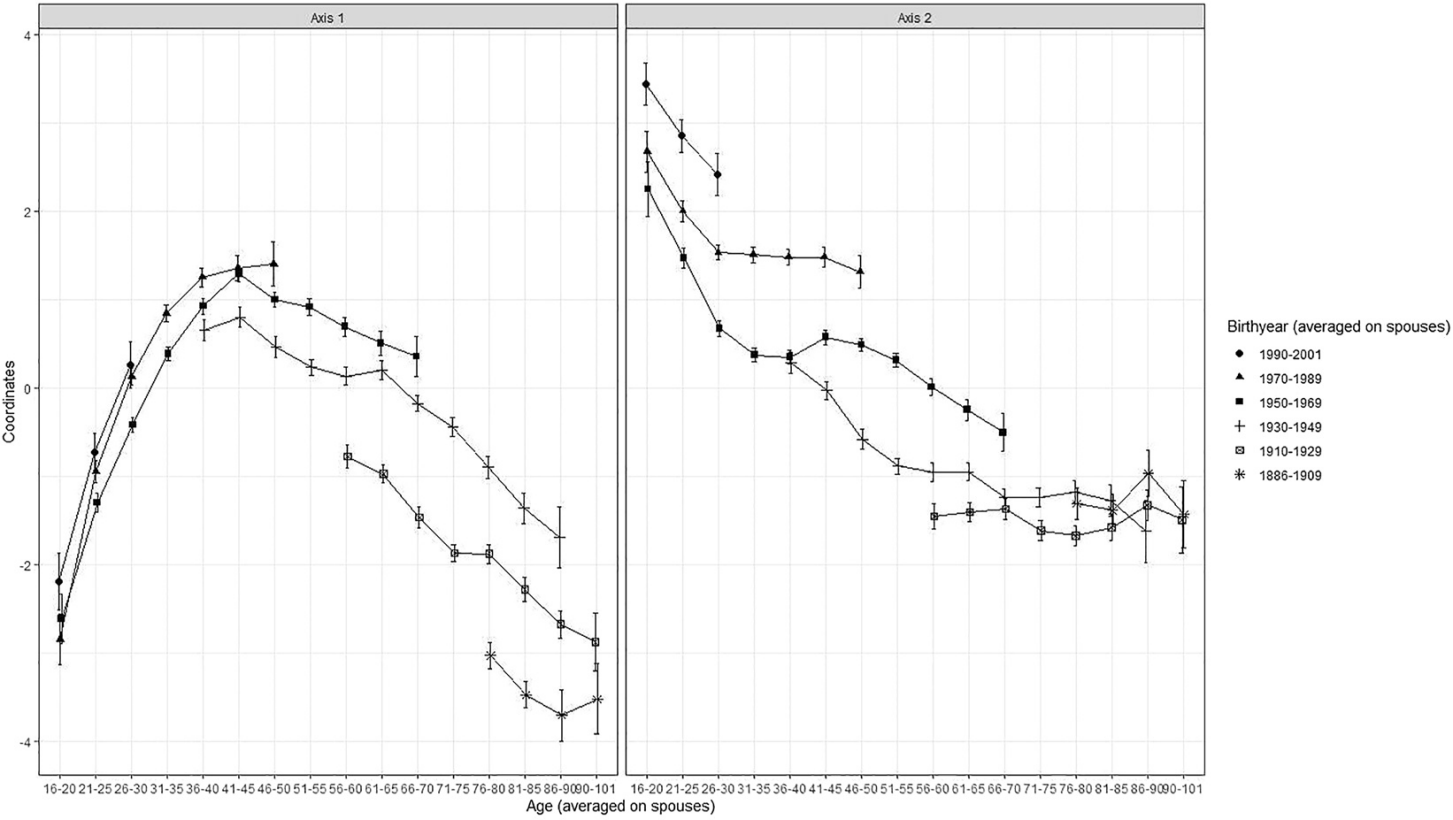
Average coordinates of households on axes 1 and 2 of the long‐term space of material consumption by age and cohort.

This evolution is associated with a generational turnover, with the gradual disappearance of the cohorts with the least integrated consumption styles. On axis 1, we observe the same inverted U‐shaped curve for each generation, with integration to consumption increasing before decreasing (Figure 6). However, the average coordinates on axis 1 increase strongly between the cohorts born before 1910 and those born after 1930. The difference in the position on axis 1 between cohorts is much smaller for those born after 1950 and insignificant after 1970. However, although it is too early to observe the evolution of the coordinates of generations born after 1990 and particularly exposed to the discourse promoting control of consumption for environmental reasons, their material consumption style before the age of 30 does not appear more frugal than that of their elders at the same age.

On axis 2, households tend to move globally toward the connected pole.^9^ This evolution seems particularly strong between 2000 and 2011 (Figure 5), a period when food produced for one's own consumption, traditional heating systems and low purchasing behavior of durable goods tended to be rarefied in favor of the development of information technology and travel, and the growth of public transport spending. This change can again be associated with that in the average position of the different generations over their life course (Figure 6). Contrary to what I observe on axis 1, generations born before 1950 occupy a stable and, at different ages, relatively similar position on axis 2, whereas the generations born after 1950 widen the gap between each other on this axis. The association with age appears reinforced by generation, with the youngest populations in 2017 occupying the highest position in this space. As on axis 1, the average position of households in 2017 is not significantly different from that in 2011, suggesting the possible end of this shift toward a more connected consumption style observed until then.

## 6 DISCUSSION AND CONCLUSION

Relying on French data, this article provides a long‐term social space analysis of material consumption styles to describe its main divides, and to measure and characterize the changes affecting them. Despite the changes in consumption—with the increasing share of services (Gershuny, 2003) and of information‐related consumption (Van Dijk, 2005)—practices once typical of the “Fordist mass consumption” era (Urry, 1990) are still a powerful structuring component of households' lifestyles.

It shows that material consumption has remained organized by structured inequalities partly related to social class between 1985 and 2017. As in other social domains (Atkinson, 2010), postmodernist theories predicting fragmentation and disorganization of material consumption (Beck, 1992; Featherstone, 2007; Giddens, 1991; Lash & Urry, 1987) are not supported by empirical evidence: material consumption styles have been structured by two highly stable divides in the recent period—excluded versus included consumers and connected versus autonomous consumption. Moreover, Gartman's view that the massification of material commodities would restore income primacy in structuring material consumption patterns (Gartman, 1991) does not fit with the empirical evidence: income is indeed a structuring variable in the material consumption space, but mainly on the first axis and not more than education, residential characteristics or age.

Nonetheless, the traditional class opposition between blue‐collar and white‐collar employment does not fit either with the data: both couples of industrial workers and of large employers, managers or professional score relatively high on the first axis—reflecting a high integration into the world of goods. In that perspective, our result support theories considering the degree of integration into mass consumption as the main divide (Bauman, 2007; Haupt, 2003; Saunders, 1986), with the first dimension of the space of material consumption being structured primarily by the opposition between included and excluded consumers. In that space, how exclusion from mass consumption overlays with other dimensions of social exclusion (economic, matrimonial, residential), whether transitory (related to certain life stages) or long‐lasting, highlights the importance of consumption in taking part in social activities (Saunders, 1986). Our results also support the importance of age and residential inequalities, with young urban households frequently having an excluded yet connected consumption style, rural families sharing the most integrated consumption and old rural households having mainly an autonomous and excluded consumption style.

However, social class has continued to structure the space of material consumption throughout the period. The divide between connected and autonomous consumption styles reflects a strong opposition in terms of professions (between managers and farmers/industrial workers), but also in terms of cultural capital. More generally, excluded‐traditional consumption styles are mainly those of households with the lowest volume of both economic and cultural capital, while included and connected consumers are found mainly among households with high volume of capital. Volume of capital—the main indicator of the position in the social space (Bourdieu, 2018; Flemmen et al., 2019)—is thus highly associated with the position in the space of material consumption, following a diagonal pattern. On the integrated‐connected quarter, capital composition distinguishes between those with a relatively high level of cultural capital whose consumption style is particularly connected, and those with a relatively high economic capital who tend to have the highest degree of integration into material consumption.

It is also worth noting that the polarities observed in the space of material consumption—as well as its relation to the social space—echo those observed for cultural consumption. Contrary to what Gartman (1991) postulated, material and non‐material cultures seem to be structured by similar inequalities. Thus, the opposition between integrated and excluded consumption on the one side and between connected and autonomous consumption on the other can be compared with that between engagement and disengagement from cultural activities and between emergent and established cultural tastes (e.g., Bennett et al., 2009; Coulangeon, 2013). Nonetheless, these similarities must not hide specificities. First, while disengagement from culture is associated with increasing participation in some in‐house practices—such as watching television—exclusion from consumption affects all the practices studied (albeit unequally). More importantly, contrary to the opposition between emergent and established repertoires, the divide between connected and autonomous consumption appears to be highly associated with education and occupation, suggesting an even stronger relationship with the position in the social space.

This study has also demonstrated the benefit of an analysis in terms of long‐term space for the analysis of social change. It highlights three simultaneous phenomena: the stability over time of the two main divides in material consumption; the shift in consumption practices contributing to such divides; and the movement of the population (in general, since households are not the same across survey waves) along these divides through the generations. The conclusion regarding social change is rather moderate: while the distribution of these consumption practices is evolving—with some becoming popularized and others becoming more divisive—these shifts do not lead to the replacement of pre‐existing divides with new ones. Instead, practices such as purchasing computer equipment become alternatively highly contributive and act to maintain the pre‐existing divides. The stability of this structure does not mean the position of households in the space is similar from year to year; rather, households tend to move toward the integrated and connected poles. This conclusion shows that Bourdieu's relational approach allows us to account for significant changes as well as stability in the social structure of lifestyles. It gives credentials to Gorski's (2013) suggestion that the central theoretical point of *Distinction* is that “the reproduction of distinctions requires the transformation of distinctions”. This long‐term analysis also corroborates the structuring nature of the dimensions organizing the space of material consumption. The notion of social structure used by scholars relying on the social space perspective since Bourdieu (Bennett et al., 2009; Lebaron, 2011) implicitly carries the ideas of durability and stability—two aspects that are empirically tested in this article.

Finally, this work intends to contribute methodologically to the comparison of spaces across time (and space), as long as the same variables are used. I propose a two‐step protocol: (1) the comparison between the axes resulting from the different annual analyses (degree of similarity, coordinates and contribution of the most contributive categories); and (2) the construction, if the similarity is high enough, of a long‐period space, using a dimensionality reduction tool (such as PCA). This method does not consider the space as stable a priori by using one of the annual spaces as an “average space” and projecting individuals from other years into it (Coulangeon, 2013). It also does not construct axes that muddle divides in each period and differences between periods by constructing a single global space and analyzing individuals from all years as active (Rosenlund, 2019). Finally, it makes it possible to establish and analyze a long‐period space while measuring changes in the practices that contribute to its construction in each period. It paves the way for analyzing the specific movements of certain social groups (e.g., urban dwellers) along the two divides studied.

This study nevertheless has some limitations. While an effort has been made to measure the occurrence or frequency of practices as well as the volumes consumed, some dimensions of consumption are left aside, in particular the usage (more or less routinized, careful, wasteful) and the precise nature of the goods and services consumed (in terms of style, quality, size). Further, the imperative to use the same variables in each period to ensure comparability spaces makes it impossible to add new, previously insignificant or unmeasured variables over the years. This could lead to an under‐estimation of the magnitude of changes affecting the structure of the material consumption space. Another limitation is the unit of analysis chosen in this work: since many consumption practices are declared at the household level, and most expenditures (apart from clothing) are not individualized in the surveys, the household was selected as the relevant unit of analysis in the analysis of social differences in material consumption. However, the different individuals who make up the household, depending on their gender or generational preferences and time availability, do not participate in the same way in the consumption of different goods and services. Also, while the consumption of French households shifted globally—especially during the 2010s—toward the most consuming and polluting poles, this ceased after 2011. A possible explanation lies in the reorientation of households' consumption toward less material forms of consumption—starting with digital and media services. Further studies are needed to assess the nature, impact and generalizability to similar countries of the shift observed in France over the last decade.

## Data Availability

The raw data that support the findings of this study are available under licence via the French Data Archives for social science. URL: https://commande.progedo.fr/en/commande/nouvelle/enquete/selection. The processed data are available from the author upon reasonable request.

## Endnotes

^1^
For a notable exception, see Rosenlund (2000, 2019).

^2^
Lifestyles also include cultural and other practices, routines and habits apart from consumption.

^3^
See Table A5 for details of categories.

^4^
Some material consumption practices are not included in the set because of their poor comparability across households or survey years, such as practices related to home furniture.

^5^
Modifying some variables, using a specific MCA or a Multiple Factor Analysis (distinguishing five groups of variables) does not change the results presented here.

^6^
Other methods can be used to compute those compromise—so‐called “long‐term”—axes. As a robustness check, we also computed them as the first two factors resulting from promax and varimax rotated PCAs on the first five factors from each of the six annual MCAs. We also computed them, in a simpler way, as the mean of annual axes 1 and as the mean of annual axes 2. Those three methods provide almost the same results as the first factors of two PCAs ran separately on the subset of annual axes 1 and on the subset of annual axes 2 (see Table A4 for Pearson's coefficient correlation between the long‐term axes computed using those different methods).

^9^
The decline in the average position on this axis between 1989 and 2000 is not easy to explain. It matches, in the data, a decline in clothing and public transport consumption specific to that year. A small bias in the data collection is suspected (see Table A5).

## Appendix

**TABLE A1:** Eigenvalues of first 10 dimensions of annual Multiple Correspondence Analysis

Dim	1985			1989			2000		
	Eigenvalue	% variance	Modified rate	Eigenvalue	% variance	Modified rate	Eigenvalue	% variance	Modified rate
1	0.118	5.42	56%	0.125	5.73	60%	0.119	5.46	56%
2	0.094	4.32	28%	0.092	4.24	24%	0.088	4.05	22%
3	0.062	2.85	6%	0.063	2.87	6%	0.072	3.29	11%
4	0.057	2.63	4%	0.060	2.74	4%	0.061	2.78	5%
5	0.054	2.47	3%	0.054	2.5	3%	0.053	2.45	3%
6	0.047	2.15	1%	0.047	2.14	1%	0.048	2.22	1%
7	0.045	2.08	1%	0.046	2.11	1%	0.046	2.11	1%
8	0.044	2.04	1%	0.044	2.02	1%	0.044	2	1%
9	0.043	1.98	1%	0.042	1.94	0%	0.042	1.94	0%
10	0.041	1.89	0%	0.042	1.91	0%	0.041	1.9	0%

Dim	2006			2011			2017		
	Eigenvalue	% variance	Modified rate	Eigenvalue	% variance	Modified rate	Eigenvalue	% variance	Modified rate
1	0.124	5.69	60%	0.124	5.67	55%	0.121	5.54	55%
2	0.091	4.2	24%	0.094	4.34	25%	0.093	4.28	25%
3	0.068	3.13	8%	0.074	3.39	10%	0.073	3.36	11%
4	0.057	2.61	3%	0.060	2.76	4%	0.056	2.55	3%
5	0.050	2.3	2%	0.053	2.42	2%	0.053	2.42	2%
6	0.047	2.16	1%	0.047	2.15	1%	0.047	2.14	1%
7	0.046	2.1	1%	0.045	2.07	1%	0.046	2.1	1%
8	0.044	2.03	1%	0.045	2.04	1%	0.045	2.04	1%
9	0.043	1.97	0%	0.044	2.03	1%	0.044	2.01	1%
10	0.042	1.91	0%	0.042	1.91	0%	0.042	1.93	0%

**TABLE A2:** Pearson's correlation between the 63,983 households' coordinates on each axis of the six annual spaces of material consumption

	Dim_1_1985	Dim_1_1989	Dim_1_2000	Dim_1_2006	Dim_1_2011	Dim_1_2017	Dim_2_1985	Dim_2_1989	Dim_2_2000	Dim_2_2006	Dim_2_2011	Dim_2_2017
Dim_1_1985	1	1.00	0.98	0.99	0.98	0.97	0.10	0.12	0.15	0.04	0.03	0.05
Dim_1_1989		1	0.99	0.99	0.99	0.98	0.06	0.08	0.13	0.01	−0.01	0.02
Dim_1_2000			1	0.99	0.99	0.98	0.01	0.04	0.07	−0.05	−0.06	−0.04
Dim_1_2006				1	0.99	0.99	0.10	0.12	0.15	0.03	0.02	0.05
Dim_1_2011					1	0.99	0.06	0.08	0.12	0.01	−0.02	0.02
Dim_1_2017						1	0.00	0.03	0.07	−0.05	−0.07	−0.03
Dim_2_1985							1	0.99	0.88	0.90	0.93	0.92
Dim_2_1989								1	0.89	0.89	0.93	0.92
Dim_2_2000									1	0.98	0.97	0.97
Dim_2_2006										1	0.98	0.98
Dim_2_2011											1	0.99
Dim_2_2017												1

**TABLE A3:** Pearson's correlation between the 89 categories' coordinates on each axis of the six annual spaces of material consumption

	Dim_1_1985	Dim_1_1989	Dim_1_2000	Dim_1_2006	Dim_1_2011	Dim_1_2017	Dim_2_1985	Dim_2_1989	Dim_2_2000	Dim_2_2006	Dim_2_2011	Dim_2_2017
Dim_1_1985	1	0.98	0.91	0.89	0.85	0.82	0.03	0.04	0.07	0.00	−0.03	−0.01
Dim_1_1989		1	0.94	0.93	0.89	0.87	−0.01	−0.01	0.04	−0.03	−0.06	−0.04
Dim_1_2000			1	0.95	0.93	0.92	−0.04	−0.04	−0.03	−0.11	−0.14	−0.11
Dim_1_2006				1	0.96	0.96	0.07	0.06	0.07	−0.03	−0.03	0.00
Dim_1_2011					1	0.97	−0.01	−0.01	0.01	−0.08	−0.10	−0.06
Dim_1_2017						1	−0.08	−0.08	−0.05	−0.16	−0.17	−0.12
Dim_2_1985							1	0.99	0.82	0.82	0.85	0.84
Dim_2_1989								1	0.84	0.84	0.87	0.86
Dim_2_2000									1	0.95	0.94	0.94
Dim_2_2006										1	0.96	0.96
Dim_2_2011											1	0.99
Dim_2_2017												1

**TABLE A4:** Pearson's correlation coefficient between long‐term axes 1 and 2 depending on the method used to compute them

	PCA on axes 1 1st axis	PCA on axes 2 1st axis
Varimax rotated PCA on 5 axes—2nd axis (PCAmixdata)	0.99874	0.01402
Promax rotated PCA on 5 axes—1st axis (psych)	0.99976	0.03929
Mean—axes 1	1.00000	0.04194
Varimax rotated PCA on 5 axes—1st axis (PCAmixdata)	0.00989	0.99709
Promax rotated PCA on 5 axes—2nd axis (psych)	0.03767	0.99911
Mean—axes 2	0.04172	1.00000

**TABLE A5:** Frequency of the 28 active variables, 1985–2017

Variables	Categories	Frequency (in per cent)						
		1985	1989	2000	2006	2011	2017	Mean
Share in in‐house food budget								
Beef and veal meat	0%–3%	31.6	37.1	58.5	57.6	60.1	63.8	51.4
	3%–8%	16.3	16	15.6	15	13.7	12.5	14.9
	8%–14%	19.6	18.8	11.6	12.1	11.2	10.8	14
	>14%	32.5	28	14.3	15.3	15	12.8	19.7
Pork and poultry meat	0%–3%	28.2	29.6	54.6	60.2	58.8	60.7	48.6
	3%–8%	17.2	17.9	18.3	17.4	15.5	16	17.1
	8%–14%	21	20.2	14.2	12.2	12.8	12	15.4
	>14%	33.6	32.4	12.9	10.2	12.9	11.3	19
Butter and cheese	0%–3%	21.5	23.4	28.2	33.7	36.9	37.2	30.1
	3%–8%	27.9	27.8	27.6	28.2	26.2	25.7	27.2
	8%–14%	30.6	29	25.3	23.3	21.5	20.8	25.1
	>14%	20	19.8	18.8	14.8	15.4	16.3	17.6
Vegetables	0%–3%	13.4	13.9	31.8	24.9	29.4	27.3	23.4
	3%–8%	22.6	23.9	31.7	27.1	25.2	24.2	25.8
	8%–14%	31.8	30.8	21.5	25.7	24.7	24.4	26.5
	>14%	32.2	31.3	15.1	22.3	20.8	24.1	24.3
Cereal products	0%–6%	13.5	11.7	9.6	11.4	13.3	15.5	12.5
	6%–10%	21.5	18.4	13.8	13	14.5	13.8	15.8
	10%–20%	45.2	45.1	42.7	38.4	36	35.9	40.6
	>20%	19.9	24.8	33.9	37.2	36.2	34.8	31.1
Diversity of food produced for own consumption	No food produced	65.8	69.9	67.2	79.3	79.3	81.6	73.9
	1 food category	14.4	13.7	16.1	10.7	10.6	10.5	12.7
	2 food categories	19.7	16.4	16.6	10	10.1	7.9	13.5
Spending on bottled water per CU	0 euros	69.2	64.7	54.4	56	64.1	64.6	62.2
	0.01–50 euros	6.6	6.8	10.7	11.4	10	10.5	9.3
	50–100 euros	7.6	9.5	12	11.6	10.1	9.9	10.1
	>100 euros	16.6	19	22.9	20.9	15.8	15	18.4
Owning								
Freezer	No	63.5	59.5	46.9	46.1	47.7	49.3	52.1
	Yes	36.5	40.5	53.1	53.9	52.3	50.7	47.9
Dishwasher	No	77.6	72.3	59.5	54.9	49.8	42	59.3
	Yes	22.4	27.7	40.5	45.1	50.2	58	40.7
Washing machine	No	17.9	15.4	9.1	8.9	7.4	5.8	10.8
	Yes	82.1	84.6	90.9	91.1	92.6	94.2	89.2
Purchasing in the last year								
IT devices	No	97.7	97.6	83.9	76.3	59.4	60.1	79.1
	Yes	2.3	2.4	16.1	23.7	40.6	39.9	20.9
Audio/video/camera devices	No	88.3	83.9	87.4	82.6	78	87.9	84.7
	Yes	11.7	16.1	12.6	17.4	22	12.1	15.3
Household appliances	No	83.6	80.1	82.1	83.1	78.8	80.1	81.3
	Yes	16.4	19.9	17.9	16.9	21.2	19.9	18.7
Acquiring second‐hand devices/appliances (last year)	No device/appliance bought	37	33.1	39.1	29.8	22.5	24.9	31.1
	None	53.7	57.3	54.6	63.1	66.2	60.9	59.3
	Partly	4.2	4.8	2.9	4.3	7.7	8.7	5.4
	Mainly	5	4.8	3.5	2.8	3.6	5.5	4.2
Extending devices/appliances lifespan	No	92.1	92	95.4	97.2	90.4	88.4	92.6
	Yes	7.9	8	4.6	2.8	9.6	11.6	7.4
Purchasing in the last 2 months (per person)								
Shoes	None	49.4	48.6	53.6	49.7	47.9	49.8	49.8
	1 or less	45	45.1	43.3	40.6	38.3	37.4	41.6
	>1	5.6	6.3	3	9.7	13.7	12.8	8.5
Pants, dress or skirt	None	46.1	46.4	51	49.1	45.6	47.2	47.6
	1 or less	43	42.2	43.4	31.9	33.3	31.8	37.6
	>1	11	11.3	5.5	19	21.1	20.9	14.8
Leisure/outwear clothes	None	59.3	54.8	67.1	66.3	68.6	68.4	64.1
	Some	40.7	45.2	32.9	33.7	31.4	31.6	35.9
Clothes repairing or maintenance practices	No	79	82.9	90.3	92.3	95.8	96.1	89.4
	Yes	21	17.1	9.7	7.7	4.2	3.9	10.6
Type of energy used for heating	Wood or coal	15.4	13.8	8.7	7.4	7.5	9.9	10.4
	Collective heating	23.9	22.7	20.9	21.2	22.8	21.2	22.1
	Electricity	18.8	22.5	23.7	24.7	30	32.3	25.3
	Fuel oil	20.3	17.4	16.8	16	11.8	9.7	15.3
	Gas or LPG	21.7	23.7	29.9	30.7	28	27	26.8
Spending in piped water per CU	<100 euros	55.9	47.3	17.1	17.5	18.4	14.4	28
	100–150 euros	24.2	26.5	24.2	24.3	21.6	18.9	23.3
	150–225 euros	13.8	16.8	30.2	28.8	28.8	29.1	24.7
	>225 euros	6.2	9.3	28.5	29.4	31.2	37.6	23.9
Estimated volume in non‐renewable energy per consumption unit	<1500 KW NCV	24.1	19.5	14	11.6	14.5	15.1	16.5
	1500–2750 KW NCV	22.7	23.8	21.1	15.9	18.5	20.1	20.4
	2750–4750 KW NCV	17	22.1	23.3	20.9	25.8	30	23.2
	4750–9000 KW NCV	14.6	15.6	18.7	26.8	26.3	23.5	20.9
	>9000 KW NCV	21.5	18.9	22.9	24.8	14.9	11.2	19
Number of cars	0	25.5	25	19.3	17.7	19.4	18.9	20.9
	1	52.6	50.9	49.9	47.9	47.5	45.7	49.1
	>1	21.9	24.2	30.9	34.4	33.1	35.4	30
Diesel engine(s)	No	94.8	87.1	52.8	41.5	31.6	28.2	52.7
	Yes	5.2	12.9	47.2	58.5	68.4	71.8	47.3
Volume of oil consumed	<50 L	48.7	50.2	49.9	56.7	55.7	58.7	53.5
	50–850 L	22	19.2	15.2	12.3	9.7	10.2	14.6
	850–1300 L	14.5	14.6	14.6	11.6	10.8	10.5	12.7
	>1300 L	14.8	16	20.2	19.4	23.7	20.6	19.2
Spending in public transportation per consumption unit	None	81	82.9	87.6	65.2	68.8	66.9	75.4
	<150 euros	3.7	3.5	2.3	11.2	8.3	9.3	6.4
	150–450 euros	7.2	6.1	3.4	9.6	8.5	8.8	7.3
	>450 euros	8	7.6	6.7	13.9	14.4	15.1	11
Trips in the last year								
Abroad	0	84	80.2	77.2	79.1	75.3	74.4	78.4
	1	12.9	15.4	16.6	16.2	18.1	17.8	16.2
	>1	3.1	4.4	6.2	4.7	6.6	7.8	5.5
Domestic	0	43.5	43.7	46.8	57	51.7	54	49.5
	1	29.5	28.4	28.9	25.3	25.6	24.4	27
	2	14.2	14.8	13.5	10.1	11.1	10.3	12.3
	>2	12.8	13.2	10.8	7.5	11.6	11.2	11.2

**TABLE A6:** Coordinates and contribution of active categories on axes 1 of the annual space of material consumption, 1985–2017

Variables	Categories	1985		1989		2000		2006		2011		2017	
		contrib.	coord.	contrib.	coord.	contrib.	coord.	contrib.	coord.	contrib.	coord.	contrib.	coord.
Share in in‐house food budget													
Beef and veal meat	0%–3%	0.33	−0.18	0.61	−0.24	0.44	−0.16	0.44	−0.16	0.66	−0.19	0.76	−0.2
	3%–8%	0.22	0.21	0.38	0.29	0.68	0.38	0.61	0.38	0.87	0.48	1.11	0.56
	8%–14%	0.19	0.18	0.09	0.13	0.31	0.3	0.41	0.35	0.49	0.39	0.55	0.42
	>14%	0.01	−0.03	0.04	0.07	0	0	0	−0.02	0.03	0.08	0.08	0.15
Pork and poultry meat	0%–3%	1.16	−0.37	1.42	−0.41	0.71	−0.21	0.52	−0.17	0.87	−0.22	0.94	−0.23
	3%–8%	0.08	0.12	0.14	0.16	0.77	0.38	0.88	0.42	0.95	0.47	1.26	0.53
	8%–14%	0.23	0.19	0.37	0.25	0.34	0.28	0.37	0.33	0.6	0.41	0.63	0.43
	>14%	0.18	0.13	0.16	0.13	0.01	0.05	0.01	−0.07	0.04	0.11	0.03	0.09
Butter and cheese	0%–3%	0.45	−0.26	0.74	−0.33	1.39	−0.4	1.09	−0.33	1.53	−0.37	1.5	−0.36
	3%–8%	0.08	0.1	0.15	0.14	0.5	0.25	0.41	0.23	0.7	0.31	0.63	0.29
	8%–14%	0.19	0.14	0.3	0.19	0.42	0.24	0.46	0.26	0.63	0.32	0.59	0.32
	>14%	0.03	−0.08	0.03	−0.07	0.02	−0.06	0.02	−0.06	0	−0.02	0.01	0.04
Vegetables	0%–3%	0.8	−0.45	0.95	−0.49	0.76	−0.28	0.69	−0.31	1.18	−0.36	1.21	−0.37
	3%–8%	0.34	0.22	0.46	0.26	1.05	0.33	0.73	0.31	0.72	0.32	0.71	0.32
	8%–14%	0.21	0.15	0.28	0.18	0.12	0.14	0.32	0.21	0.45	0.25	0.67	0.31
	>14%	0.13	−0.11	0.21	−0.15	0.39	−0.29	0.43	−0.26	0.09	−0.12	0.18	−0.16
Cereal products	0%–6%	0.05	−0.11	0.21	−0.25	0.4	−0.37	0.51	−0.4	0.15	−0.2	0.37	−0.29
	6%–10%	0.11	0.13	0.04	0.09	0.05	0.11	0.01	0.04	0.08	0.14	0.26	0.26
	10%–20%	0.04	0.06	0.27	0.14	0.56	0.21	0.44	0.2	0.84	0.29	0.71	0.26
	>20%	0.24	−0.2	0.32	−0.21	0.42	−0.2	0.1	−0.1	0.69	−0.25	0.55	−0.22
Diversity of food produced for own consumption	no food produced	0.7	−0.19	0.44	−0.15	0.56	−0.17	0.21	−0.1	0.39	−0.13	0.44	−0.13
	1 food category	0.55	0.36	0.51	0.36	0.43	0.3	0.38	0.35	0.85	0.53	1.1	0.59
	2 food categories	0.79	0.36	0.53	0.34	0.74	0.39	0.43	0.38	0.65	0.47	0.84	0.6
Spending on bottled water per CU	0 euros	0.02	−0.03	0.26	−0.12	0.96	−0.24	0.79	−0.22	0.57	−0.18	0.59	−0.18
	0.01–50 euros	0.03	0.13	0.12	0.25	0.21	0.26	0.47	0.38	0.39	0.37	0.29	0.3
	50–100 euros	0.05	0.14	0.48	0.42	0.52	0.38	0.58	0.42	0.34	0.34	0.65	0.47
	>100 euros	0	0	0.06	0.11	0.45	0.26	0.15	0.16	0.32	0.26	0.24	0.23
Possession													
Freezer	No	2.38	−0.35	3.01	−0.42	3.41	−0.49	2.77	−0.46	2.52	−0.43	2.62	−0.42
	Yes	4.19	0.62	4.45	0.62	3.01	0.43	2.37	0.39	2.29	0.39	2.55	0.41
Dishwasher	No	1.76	−0.27	2.44	−0.34	3.94	−0.47	3.95	−0.5	4.77	−0.58	5.69	−0.68
	Yes	6.17	0.96	6.4	0.9	5.78	0.69	4.82	0.61	4.73	0.57	4.11	0.49
Washing machine	No	7.53	−1.18	7.03	−1.26	4.99	−1.35	3.56	−1.18	3.38	−1.26	2.85	−1.29
	Yes	1.64	0.26	1.28	0.23	0.5	0.14	0.35	0.12	0.27	0.1	0.18	0.08
Purchase during last year													
IT devices	No	0.02	−0.03	0.01	−0.02	0.39	−0.12	0.6	−0.16	1.77	−0.32	1.57	−0.3
	Yes	0.91	1.15	0.47	0.84	2.01	0.64	1.92	0.53	2.59	0.47	2.36	0.45
Audio/video/camera devices	No	0.05	−0.04	0.18	−0.09	0.1	−0.06	0.23	−0.1	0.31	−0.12	0.09	−0.06
	Yes	0.37	0.33	0.92	0.45	0.72	0.44	1.07	0.46	1.1	0.42	0.63	0.42
Household appliances	No	0.3	−0.11	0.38	−0.13	0.31	−0.11	0.24	−0.1	0.37	−0.13	0.3	−0.11
	Yes	1.54	0.56	1.53	0.52	1.43	0.52	1.17	0.49	1.39	0.48	1.19	0.45
Acquiring second‐hand devices/appliances (last year)	No device/appliance bought	3.56	−0.56	3.83	−0.63	3.7	−0.56	4.71	−0.74	5.25	−0.9	4.47	−0.78
	None	1.81	0.33	1.72	0.32	2.37	0.38	1.74	0.31	1.21	0.25	1	0.24
	Partly	0.61	0.7	0.58	0.65	0.26	0.54	0.66	0.73	0.46	0.46	0.91	0.6
	Mainly	0	0.04	0.02	−0.12	0.01	−0.11	0.03	−0.2	0	0	0	−0.02
Extending devices/appliances lifespan	No	0.8	0.58	0.68	0.55	0.42	0.55	0.3	0.6	1.54	0.74	1.24	0.6
	Yes	0.07	−0.05	0.06	−0.05	0.02	−0.03	0.01	−0.02	0.16	−0.08	0.16	−0.08
Purchase during the last 2 months (per person)													
Shoes	None	2.93	−0.44	2.62	−0.43	1.53	−0.31	2.94	−0.45	2.65	−0.44	2.35	−0.4
	1 or less	2.75	0.45	2.4	0.43	1.97	0.39	2.24	0.44	1.78	0.4	1.98	0.42
	>1	0.15	0.3	0.12	0.26	0.01	−0.11	0.67	0.49	0.66	0.41	0.37	0.31
Pants, dress or skirt	None	3.51	−0.5	3.23	−0.49	1.97	−0.36	3.5	−0.5	2.82	−0.46	2.49	−0.42
	1 or less	2.61	0.45	2.67	0.47	2.21	0.41	2.18	0.49	1.45	0.39	1.71	0.43
	>1	0.41	0.35	0.23	0.27	0.01	0.08	1.2	0.47	0.91	0.39	0.58	0.3
Leisure/outwear clothes	None	2.43	−0.37	2.42	−0.39	1.08	−0.23	1.68	−0.3	1.36	−0.26	1.22	−0.25
	Some	3.54	0.54	2.93	0.48	2.21	0.47	3.29	0.58	2.96	0.57	2.65	0.53
Clothes repairing or maintenance practices	No	0.26	−0.1	0.14	−0.08	0.05	−0.04	0.03	−0.03	0.01	−0.02	0.01	−0.02
	Yes	0.99	0.39	0.68	0.37	0.45	0.39	0.32	0.38	0.32	0.52	0.24	0.45
Type of energy used for heating	Wood or coal	0.58	−0.35	0.2	−0.22	0	−0.02	0.03	0.11	0.25	0.34	1.13	0.62
	Collective heating	0.96	−0.37	1.28	−0.44	2.24	−0.6	1.74	−0.53	1.92	−0.54	2.31	−0.61
	Electricity	0.06	0.1	0.07	0.11	0	0.01	0	0.01	0	0.02	0.02	−0.05
	Fuel oil	1.03	0.41	1.11	0.47	0.93	0.43	0.59	0.36	0.69	0.45	0.5	0.42
	Gas or LPG	0.21	0.18	0.08	0.11	0.27	0.17	0.19	0.15	0.15	0.14	0.2	0.16
Spending in piped water per CU	<100 euros	0.56	−0.16	0.84	−0.21	0.02	−0.08	0.04	−0.11	0.01	0.06	0.16	0.24
	100–150 euros	0.82	0.43	0.81	0.4	0.1	−0.13	0.7	0.36	0.56	0.38	0.43	0.34
	150–225 euros	0.52	0.45	0.53	0.41	0.01	−0.03	0.09	0.1	0.82	−0.28	0.35	0.23
	>225 euros	0.19	0.41	0.24	0.37	0.23	0.16	0.49	−0.21	0.13	0.11	0.73	−0.21
Estimated volume in non‐renewable energy per consumption unit	<1500 KW NCV	1.27	−0.42	1.42	−0.5	1.22	−0.54	0.93	−0.53	1.16	−0.53	1.68	−0.61
	1500–2750 KW NCV	0.2	−0.17	0.17	−0.16	0.46	−0.27	0.4	−0.29	0.86	−0.4	0.21	−0.19
	2750–4750 KW NCV	0.02	0.06	0.06	0.1	0.06	0.1	0.03	0.07	0.33	0.21	0.42	0.22
	4750–9000 KW NCV	0.49	0.33	0.44	0.31	0.08	0.12	0.28	0.19	0.51	0.26	0.28	0.2
	>9000 KW NCV	0.91	0.37	0.65	0.35	0.99	0.38	0.2	0.17	0.15	0.19	0.08	0.16
Number of cars	0	9.79	−1.13	8.57	−1.1	8.58	−1.22	6.99	−1.17	6.46	−1.07	6.37	−1.07
	1	0.35	0.15	0.1	0.08	0.09	−0.08	0.46	−0.18	0.23	−0.13	0.62	−0.21
	>1	6.04	0.96	6.38	0.96	7.22	0.88	7.27	0.86	6.36	0.82	7.51	0.85
Diesel engine(s)	No	0.03	−0.03	0.16	−0.08	2.96	−0.4	4.57	−0.57	3.46	−0.6	2.81	−0.58
	Yes	0.87	0.94	1.99	0.97	4.6	0.63	4.41	0.55	1.71	0.3	1.1	0.23
Volume of oil consumed	<50 L	5.02	−0.56	4.22	−0.52	3.73	−0.49	2.76	−0.41	2.34	−0.38	2.56	−0.38
	50–850 L	2.59	0.64	2.61	0.71	0.95	0.46	0.94	0.52	0.43	0.4	0.85	0.53
	850–1300 L	1.23	0.55	1.06	0.54	1.43	0.57	1.24	0.61	0.7	0.48	1.26	0.64
	>1300 L	1.62	0.63	1.38	0.57	1.48	0.5	1.52	0.52	1.9	0.53	1.62	0.52
Spending in public transportation per consumption unit	None	0	−0.01	0	−0.01	0.01	0.02	0.01	−0.02	0.01	−0.02	0	−0.01
	<150 euros	0.18	0.4	0.08	0.29	0.03	0.22	0.28	0.3	0.54	0.47	0.65	0.49
	150–450 euros	0	−0.03	0	0.05	0	−0.04	0.01	0.07	0.01	0.06	0.07	0.17
	>450 euros	0.02	−0.1	0.01	−0.06	0.13	−0.26	0.16	−0.2	0.16	−0.19	0.49	−0.33
Trips during the last year													
Abroad	0	0.06	−0.05	0.09	−0.06	0.23	−0.1	0.21	−0.1	0.15	−0.08	0.15	−0.08
	1	0.27	0.26	0.23	0.23	0.41	0.29	0.61	0.36	0.29	0.23	0.23	0.21
	>1	0.03	0.19	0.12	0.31	0.42	0.48	0.17	0.36	0.19	0.31	0.21	0.3
Domestic	0	1.92	−0.38	1.64	−0.36	1.47	−0.32	1.11	−0.26	1.61	−0.33	1.18	−0.27
	1	0.37	0.2	0.24	0.17	0.25	0.17	0.48	0.26	0.77	0.32	0.67	0.3
	2	0.52	0.35	0.69	0.4	0.68	0.41	0.67	0.48	0.7	0.47	0.46	0.39
	>2	0.75	0.44	0.53	0.37	0.61	0.43	0.48	0.47	0.31	0.31	0.28	0.29

**TABLE A7:** Coordinates and contribution of active categories on axes 2 of the annual space of material consumption, 1985–2017

Variables	Categories	1985		1989		2000		2006		2011		2017	
		contrib.	coord.	contrib.	coord.	contrib.	coord.	contrib.	coord.	contrib.	coord.	contrib.	coord.
Share in in‐house food budget													
Beef and veal meat	0%–3%	0.78	0.25	0.77	0.23	0.06	0.05	0.03	0.03	0.17	0.09	0.05	0.05
	3%–8%	0.05	−0.09	0.1	−0.13	0.04	0.08	0.04	0.08	0.01	−0.03	0	0.02
	8%–14%	0.21	−0.17	0.1	−0.12	0	0.01	0	−0.02	0.04	−0.09	0.01	−0.04
	>14%	0.13	−0.1	0.27	−0.16	0.52	−0.3	0.22	−0.19	0.37	−0.26	0.26	−0.23
Pork and poultry meat	0%–3%	1.58	0.38	1.91	0.41	0	−0.01	0.01	0.02	0.12	0.07	0.06	0.05
	3%–8%	0.01	0.04	0.01	−0.03	0.16	0.15	0.05	0.09	0.01	−0.03	0	0
	8%–14%	0.05	−0.08	0.08	−0.1	0	0.03	0	0.01	0.1	−0.15	0.04	−0.1
	>14%	1.14	−0.3	1.11	−0.3	0.17	−0.18	0.27	−0.26	0.13	−0.17	0.13	−0.17
Butter and cheese	0%–3%	1.02	0.35	0.79	0.29	0.02	0.04	0.07	0.07	0.34	0.15	0.2	0.11
	3%–8%	0.12	−0.11	0.1	−0.1	0.18	0.13	0.04	0.06	0	−0.01	0	0.01
	8%–14%	0.31	−0.16	0.13	−0.11	0.01	0.03	0	−0.02	0.12	−0.12	0.05	−0.08
	>14%	0	0.01	0.03	−0.06	0.6	−0.28	0.38	−0.26	0.23	−0.2	0.23	−0.19
Vegetables	0%–3%	1.94	0.62	2.55	0.69	0.55	−0.2	0.41	−0.2	0	0	0.02	−0.04
	3%–8%	0.01	0.04	0.01	0.04	0.07	0.08	0.01	−0.03	0.08	−0.09	0.06	−0.08
	8%–14%	0.05	−0.07	0.04	−0.06	0.14	0.13	0.09	0.09	0	−0.02	0	0
	>14%	0.59	−0.22	0.92	−0.27	0.07	0.11	0.25	0.17	0.14	0.13	0.16	0.13
Cereal products	0%–6%	0.34	0.26	0.01	0.04	0	0.01	0.02	0.07	0.01	0.04	0.1	0.13
	6%–10%	0.16	−0.14	0.32	−0.21	0.04	−0.08	0.01	−0.05	0.1	−0.14	0.01	−0.04
	10%–20%	0.31	−0.13	0.31	−0.13	0.03	0.04	0	0	0.11	−0.09	0.15	−0.11
	>20%	0.62	0.29	1.4	0.38	0.01	−0.02	0	−0.01	0.21	0.12	0.06	0.06
Diversity of food produced for own consumption	no food produced	4.16	0.41	3.64	0.37	2.82	0.32	1.62	0.23	1.36	0.21	0.89	0.17
	1 food category	1.5	−0.52	1.95	−0.61	1.72	−0.51	2.37	−0.75	1.63	−0.64	1.61	−0.63
	2 food categories	7.17	−0.98	7.12	−1.06	4.36	−0.81	3.98	−1.01	3.82	−1	2.46	−0.9
Spending on bottled water per CU	0 euros	0	0.01	0.01	0.01	0.17	−0.09	0.01	−0.02	0.01	0.02	0	0.01
	0.01–50 euros	0.11	0.21	0.1	0.2	0.29	0.26	0.33	0.27	0.17	0.21	0.24	0.24
	50–100 euros	0.07	0.15	0.01	0.06	0.13	0.17	0	0.01	0.02	−0.07	0.01	0.06
	>100 euros	0.26	−0.2	0.16	−0.15	0	0	0.08	−0.1	0.18	−0.17	0.38	−0.26
Possession													
Freezer	No	1.9	0.28	1.61	0.26	3.39	0.42	4.1	0.48	4.06	0.47	3.6	0.44
	Yes	3.34	−0.49	2.39	−0.39	2.99	−0.37	3.51	−0.41	3.7	−0.43	3.5	−0.42
Dishwasher	No	0.06	−0.05	0.04	−0.04	0.05	0.05	0.23	0.1	0.5	0.16	0.58	0.19
	Yes	0.22	0.16	0.1	0.1	0.08	−0.07	0.28	−0.13	0.5	−0.16	0.42	−0.14
Washing machine	No	0.97	0.38	1.06	0.42	0.87	0.49	0.95	0.52	1.83	0.81	1.01	0.67
	Yes	0.21	−0.08	0.19	−0.08	0.09	−0.05	0.09	−0.05	0.15	−0.06	0.06	−0.04
Purchase during last year													
IT devices	No	0.02	−0.02	0.01	−0.02	0.56	−0.13	1.41	−0.22	2.92	−0.36	2.61	−0.34
	Yes	0.65	0.87	0.52	0.75	2.91	0.67	4.52	0.7	4.27	0.53	3.92	0.51
Audio/video/camera devices	No	0.05	−0.04	0.18	−0.07	0.19	−0.07	0.29	−0.09	0.17	−0.08	0.06	−0.04
	Yes	0.36	0.29	0.94	0.39	1.3	0.51	1.36	0.45	0.6	0.27	0.41	0.3
Household appliances	No	0.12	−0.06	0.13	−0.07	0.25	−0.09	0.09	−0.05	0.15	−0.07	0.16	−0.07
	Yes	0.64	0.32	0.54	0.27	1.12	0.39	0.44	0.26	0.56	0.26	0.64	0.29
Acquiring second‐hand devices/appliances (last year)	No device/appliance bought	1.73	−0.35	2.64	−0.45	3	−0.44	3.64	−0.56	4.16	−0.7	4.56	−0.69
	None	0.57	0.17	0.6	0.16	1.37	0.25	0.91	0.19	0.4	0.13	0.36	0.12
	Partly	0.51	0.57	0.77	0.64	0.72	0.79	1	0.77	1.86	0.8	2.23	0.82
	Mainly	0.23	0.35	0.54	0.54	0.15	0.33	0.21	0.44	0.14	0.32	0.45	0.46
Extending devices/appliances lifespan	No	1.16	0.62	0.46	0.39	0.35	0.43	0.15	0.37	1.37	0.61	2.19	0.7
	Yes	0.1	−0.05	0.04	−0.03	0.02	−0.02	0	−0.01	0.15	−0.07	0.29	−0.09
Purchase during the last 2 months (per person)													
Shoes	None	2.43	−0.36	2.71	−0.38	1.89	−0.29	1.82	−0.31	2.49	−0.37	3.03	−0.4
	1 or less	1.22	0.27	1.28	0.27	1.68	0.31	0.8	0.22	0.53	0.19	0.96	0.26
	>1	2.25	1.03	2.4	1	0.77	0.79	1.49	0.63	2.98	0.76	3.09	0.79
Pants, dress or skirt	None	1.97	−0.34	2.31	−0.36	2	−0.31	1.78	−0.3	2.77	−0.4	2.95	−0.4
	1 or less	0.39	0.15	0.55	0.18	1.52	0.29	0.26	0.14	0.2	0.13	0.23	0.14
	>1	2.68	0.8	2.72	0.79	0.7	0.56	2.22	0.55	3.53	0.66	3.93	0.7
Leisure/outwear clothes	None	1.82	−0.28	2.46	−0.34	1.08	−0.2	1.49	−0.24	1.76	−0.26	2.21	−0.29
	Some	2.65	0.41	2.98	0.41	2.2	0.41	2.93	0.47	3.84	0.57	4.78	0.63
Clothes repairing or maintenance practices	No	0.3	−0.1	0.11	−0.06	0.05	−0.04	0.02	−0.02	0.01	−0.01	0	−0.01
	Yes	1.13	0.38	0.56	0.29	0.48	0.35	0.18	0.25	0.13	0.29	0.11	0.27
Type of energy used for heating	Wood or coal	4.03	−0.83	4.46	−0.91	1.87	−0.73	1.99	−0.83	1.68	−0.77	1.45	−0.62
	Collective heating	9.28	1.01	7.47	0.92	4.89	0.76	5.81	0.84	3.15	0.6	3.12	0.62
	Electricity	0.02	0.06	0.2	0.15	0.93	0.31	0.36	0.19	0.59	0.23	0.44	0.19
	Fuel oil	4.97	−0.8	5.13	−0.87	13.04	−1.39	7.63	−1.1	6.74	−1.23	6.36	−1.31
	Gas or LPG	0.26	0.18	0.2	0.15	0.54	0.21	0.02	0.04	0	−0.01	0	−0.02
Spending in piped water per CU	<100 euros	0.91	0.18	1.24	0.22	2.24	−0.67	1.96	−0.62	1.03	−0.46	0.57	−0.39
	100–150 euros	1.29	−0.48	1.04	−0.39	0.97	−0.34	0.4	−0.23	1.41	−0.52	1.05	−0.47
	150–225 euros	0.71	−0.47	0.79	−0.43	1.35	0.3	0.36	−0.18	0.53	0.2	0.11	−0.11
	>225 euros	0.51	−0.6	0.51	−0.47	0.23	0.14	2.97	0.43	0.35	0.15	1.01	0.22
Estimated volume in non‐renewable energy per consumption unit	<1500 KW NCV	4.17	0.67	3.17	0.65	1.54	0.52	2.55	0.75	3.17	0.76	2.26	0.62
	1500–2750 KW NCV	0.24	0.17	0.66	0.27	1.68	0.44	1.97	0.56	1.33	0.44	1.48	0.44
	2750–4750 KW NCV	0.01	−0.04	0	0.01	1.23	0.36	0.68	0.29	0.3	0.18	0.22	0.14
	4750–9000 KW NCV	0.08	−0.12	0.12	−0.14	0.09	0.11	0.07	−0.08	0.79	−0.28	1.19	−0.36
	>9000 KW NCV	5.5	−0.82	5.93	−0.9	13.03	−1.19	7.22	−0.86	6.7	−1.09	6.54	−1.23
Number of cars	0	0	−0.02	0.01	−0.04	0.26	0.18	1.16	0.41	0.9	0.35	1.56	0.46
	1	0.07	0.06	0.01	0.03	0	0	0.05	−0.05	0.01	−0.02	0.11	−0.08
	>1	0.12	−0.12	0	−0.02	0.15	−0.11	0.27	−0.14	0.38	−0.17	0.29	−0.15
Diesel engine(s)	No	0	0	0	0	0.23	0.1	0.42	0.15	0.03	−0.05	0.01	−0.02
	Yes	0	0.03	0	0.02	0.35	−0.15	0.41	−0.14	0.01	0.02	0	0.01
Volume of oil consumed	<50 L	0	−0.01	0.02	−0.03	0	0.01	0.08	0.06	0.12	0.08	0.05	0.05
	50–850 L	0.14	−0.13	0.05	−0.09	0	0	0	0.02	0	0	0.15	0.2
	850–1300 L	0	0.01	0.01	−0.05	0.07	−0.11	0.09	−0.14	0.04	−0.1	0.02	−0.06
	>1300 L	0.26	0.23	0.38	0.26	0.02	0.05	0.09	−0.11	0.17	−0.14	0.32	−0.2
Spending in public transportation per consumption unit	None	0.98	−0.18	0.81	−0.16	0.57	−0.13	3.47	−0.37	2.56	−0.31	3.07	−0.35
	<150 euros	0.37	0.52	0.64	0.69	0.47	0.71	0.4	0.3	0.28	0.3	0.35	0.32
	150–450 euros	1.47	0.73	1.17	0.71	0.93	0.82	1.86	0.7	1.14	0.6	1.3	0.62
	>450 euros	2.46	0.9	2.17	0.86	2.67	0.99	5.41	1	5.15	0.97	5.53	0.98
Trips during the last year													
Abroad	0	0.35	−0.11	0.52	−0.13	0.54	−0.13	0.54	−0.13	0.86	−0.17	0.95	−0.18
	1	1.08	0.47	1.19	0.45	0.81	0.35	1.08	0.41	1.47	0.46	1.54	0.48
	>1	0.94	0.89	1.07	0.79	1.24	0.7	1.15	0.79	1.24	0.7	1.28	0.66
Domestic	0	2.79	−0.41	2.65	−0.4	1.82	−0.31	1.15	−0.23	1.01	−0.23	0.83	−0.2
	1	0.01	0.02	0.06	0.07	0.14	0.11	0.31	0.18	0.07	0.09	0.09	0.1
	2	1.08	0.45	0.85	0.38	0.93	0.41	0.71	0.42	0.39	0.31	0.38	0.31
	>2	3.52	0.85	2.65	0.72	1.26	0.54	0.91	0.56	1.23	0.53	0.92	0.46

**TABLE A8:** Coordinates of active categories on axes 1 and 2 of the long‐term space of material consumption, 1985–2017

Variables	Categories	Average coordinate on long term	
		Axis 1	Axis 2
Share in in‐house food budget			
Beef and veal meat	0%–3%	−0.3	0.31
	3%–8%	0.88	−0.09
	8%–14%	0.56	−0.28
	>14%	−0.07	−0.59
Pork and poultry meat	0%–3%	−0.41	0.35
	3%–8%	0.82	0.01
	8%–14%	0.64	−0.27
	>14%	0.02	−0.74
Butter and cheese	0%–3%	−0.67	0.48
	3%–8%	0.52	−0.04
	8%–14%	0.52	−0.26
	>14%	−0.15	−0.44
Vegetables	0%–3%	−0.67	0.23
	3%–8%	0.74	−0.09
	8%–14%	0.46	−0.05
	>14%	−0.46	−0.11
Cereal products	0%–6%	−0.64	0.24
	6%–10%	0.23	−0.35
	10%–20%	0.45	−0.24
	>20%	−0.3	0.36
Diversity of food produced for own consumption	no food produced	−0.32	0.69
	1 food category	0.93	−1.5
	2 food categories	0.87	−2.36
Spending on bottled water per CU	0 euros	−0.39	0.01
	0.01–50 euros	0.79	0.6
	50–100 euros	0.94	0.15
	>100 euros	0.41	−0.41
Possession			
Freezer	No	−1.06	0.88
	Yes	1.18	−0.96
Dishwasher	No	−1.16	0.03
	Yes	1.7	−0.04
Washing machine	No	−3.14	1.1
	Yes	0.39	−0.13
Purchase during last year			
IT devices	No	−0.41	−0.46
	Yes	1.59	1.76
Audio/video/camera devices	No	−0.19	−0.17
	Yes	1.09	0.95
Household appliances	No	−0.28	−0.17
	Yes	1.26	0.77
Acquiring second‐hand devices/appliances (last year)	No device/appliance bought	−1.71	−1.34
	None	0.78	0.45
	Partly	1.58	1.97
	Mainly	−0.16	1.01
Extending devices/appliances lifespan	No	1.53	1.46
	Yes	−0.12	−0.12
Purchase during the last 2 months (per person)			
Shoes	None	−1	−0.87
	1 or less	1.01	0.59
	>1	0.93	2.21
Pants, dress or skirt	None	−1.11	−0.87
	1 or less	1.03	0.38
	>1	0.93	1.84
Leisure/outwear clothes	None	−0.69	−0.63
	Some	1.23	1.13
Clothes repairing or maintenance practices	No	−0.09	−0.06
	Yes	0.74	0.53
Type of energy used for heating	Wood or coal	−0.04	−1.89
	Collective heating	−1.25	1.88
	Electricity	0.13	0.54
	Fuel oil	0.92	−2.73
	Gas or LPG	0.39	0.23
Spending in piped water per CU	<100 euros	0	−1.13
	100–150 euros	0.76	−0.98
	150–225 euros	0.8	−0.54
	>225 euros	0.86	−0.36
Estimated volume in non‐renewable energy per consumption unit	<1500 KW NCV	−1.31	1.53
	1500–2750 KW NCV	−0.61	0.87
	2750–4750 KW NCV	0.38	0.45
	4750–9000 KW NCV	0.63	−0.27
	>9000 KW NCV	0.62	−2.5
Number of cars	0	−2.76	0.42
	1	−0.17	−0.05
	>1	2.2	−0.21
Diesel engine(s)	No	−0.03	−0.23
	Yes	1.62	0.03
Volume of oil consumed	<50 L	−1.07	0.08
	50–850 L	1.21	−0.16
	850–1300 L	1.34	−0.25
	>1300 L	1.39	0.05
Spending in public transportation per consumption unit	None	−0.05	−0.62
	<150 euros	1.07	1.19
	150–450 euros	0.19	1.73
	>450 euros	−0.38	2.41
Trips during the last year			
Abroad	0	−0.21	−0.35
	1	0.69	1.09
	>1	0.92	1.87
Domestic	0	−0.74	−0.65
	1	0.55	0.2
	2	0.95	0.86
	>2	0.89	1.46

**TABLE A9:** Coordinates and typicality tests of supplementary categories on axes 1 and 2 of the long‐term space of material consumption, 1985–2017

Variable	Category	Axis 1		Axis 2	
		Coord.	Typic.	Coord.	Typic.
Age (averaged over spouses)	16–28 years old	−0.82	−29.2	1.75	63.9
	29–38 years old	0.60	30.5	0.89	45.5
	39–48 years old	1.07	53.2	0.53	27.0
	49–58 years old	0.54	26.2	−0.26	−12.8
	59–68 years old	−0.17	−7.4	−0.97	−43.5
	69–78 years old	−1.14	−43.3	−1.38	−53.6
	79–102 years old	−2.34	−66.8	−1.39	−40.4
	*Eta2*	16%		18%	
Household type	Couple with more than two children	1.58	45.4	0.44	12.9
	Couple with two children	1.86	75.8	0.48	19.8
	Couple with a child	1.27	50.6	0.16	6.5
	Couple without children	0.32	19.9	−0.77	−49.0
	Mother with children	−0.24	−5.6	1.24	29.9
	Father with children	−0.01	−0.2^NS^	0.42	4.7
	Single woman	−2.00	−97.9	0.04	1.8^NS^
	Single man	−1.92	−72.7	0.26	9.9
	Others	−0.18	−4.0	−0.32	−7.3
	*Eta2*	34%		5%	
Highest diploma	Primary education or less	−1.24	−85.7	−1.09	−77.1
	Middle school or vocational ed.	0.35	23.5	−0.22	−15.0
	High‐school cert.	0.51	22.4	0.61	27.3
	Two year university degree	1.00	32.9	0.93	31.1
	Bachelor's degree or more	0.85	36.3	1.51	65.7
	*Eta2*	12%		15%	
Housing size	0–50 m^2^	−2.14	−99.4	1.16	54.7
	50–70 m^2^	−0.89	−45.4	0.47	24.1
	70–90 m^2^	0.09	5.0	−0.11	−6.6
	90–130 m^2^	1.13	71.1	−0.60	−38.4
	>130 m^2^	1.88	70.4	−0.68	−26.1
	*Eta2*	28%		7%	
Years spent in the housing	[0,3]	−0.22	−12.1	1.21	68.7
	(3,7]	0.35	16.4	0.56	26.7
	(7,14]	0.51	24.0	0.12	5.7
	(14,26]	0.37	17.8	−0.64	−31.8
	(26,111]	−0.53	−26.0	−1.65	−82.7
	*Eta2*	3%		17%	
Living standard percentile	1–10th	−1.23	−42.4	−0.15	−5.1
	11–25th	−0.98	−42.8	−0.46	−20.3
	26–50th	−0.29	−17.1	−0.30	−18.5
	50–75th	0.44	26.4	0.07	4.1
	76–90th	0.85	36.9	0.48	21.1
	91–99th	1.11	36.3	0.75	25.0
	100th	0.99	10.4	1.02	10.9
	*Eta2*	10%		3%	
Housing status	Social housing	−1.04	−47.3	1.21	56.1
	Tenant	−0.82	−46.1	0.99	56.8
	First‐time homebuyer	1.59	84.9	0.15	8.3
	Homeowner	0.21	16.2	−1.24	−95.3
	Free housing	−0.91	−23.6	−0.40	−10.6
	*Eta2*	15%		18%	
Residential area	Rural area	0.68	39.4	−1.65	−98.0
	Less than 20,000 residents	0.37	17.2	−0.65	−30.8
	20,000 to 100,000 residents	−0.04	−1.6^NS^	0.08	3.2
	More than 100,000 residents	−0.31	−21.1	0.73	50.0
	Paris area	−0.75	−35.0	1.67	79.0
	*Eta2*	4%		24%	
Housing type	More than two‐dwelling building	−1.10	−90.4	1.57	131.7
	Two‐dwelling building	−0.75	−17.5	0.15	3.5
	Detached house	0.82	96.9	−1.09	−131.1
	*Eta2*	15%		28%	
Household occupational category	Couple of large employers, managers/professionals mainly	1.98	50.0	1.26	32.4
	Intermediate occupations (or managers) mainly	0.87	36.9	0.74	32.0
	Clerical worker (or intermediate occupations) mainly	0.49	26.9	0.33	18.5
	Self‐employed mainly	−0.02	−0.6^NS^	−0.94	−27.0
	Couple of industrial workers mainly	0.76	28.5	−0.40	−15.5
	Monoactive clerical or industrial worker	−1.12	−77.2	−0.08	−5.4
	Agricultural	−0.37	−10.0	−2.36	−65.3
	Inactive only	−2.35	−41.6	1.23	22.1
	*Eta2*	17%		12%	
Household employment status	Working only	0.67	62.1	0.68	64.1
	Working with inactive person	0.98	38.2	−0.23	−9.3
	Unemployed with working person	0.91	19.2	0.62	13.2
	Unemployed without working person	−1.37	−32.9	0.23	5.7
	At least one student	−1.92	−31.3	2.71	45.0
	Retired only	−1.10	−64.9	−1.21	−72.8
	Other inactive	−0.94	−27.7	−1.04	−31.4
	*Eta2*	15%		15%	

*Note*: “^NS^” accounts for *p*‐values for typicality test statistics that are above the 0.01 threshold.
